# Lipid droplet–mitochondria contact sites as druggable spatial-pharmacology targets in respiratory disease: cross–cell-type mechanisms and translational strategies

**DOI:** 10.3389/fcell.2026.1935993

**Published:** 2026-09-07

**Authors:** Shuting Li, Rongrong Bai

**Affiliations:** The Second Affiliated Hospital of Nanjing University of Traditional Chinese Medicine (Jiangsu Second Hospital of Traditional Chinese Medicine), Nanjing, Jiangsu, China

**Keywords:** chronic obstructive pulmonary disease, idiopathic pulmonary fibrosis, inhaled lipid nanoparticles, lipid droplet–mitochondria contact sites, lung adenocarcinoma, peridroplet mitochondria, PFKL–PLIN2–CPT1A, PLIN5

## Abstract

Lipid droplets and mitochondria form regulated contact sites whose molecular composition, phosphorylation state and spatial geometry now behave as measurable, druggable variables in living cells. The molecular toolkit has been mapped in liver, muscle and adipose tissue, yet its application to respiratory disease has lagged despite a lung-specific dependence on lipid biology that spans surfactant biogenesis, alveolar-macrophage lipid handling and fibroblast-driven remodelling. This review argues that the lipid-droplet–mitochondria contact site is an emerging spatial-pharmacology target in respiratory medicine, and organises the evidence into a three-way matrix with explicit grading of direct, indirect and cross-tissue evidence. A common molecular toolkit built around perilipin-family scaffolds, mitochondrial-outer-membrane tethers, an endoplasmic-reticulum bridging apparatus and the PFKL–PLIN2–CPT1A flux node (mechanistically established in hepatocellular carcinoma and inferred in lung) is rewired into cell-type-specific configurations across alveolar type 2 cells, macrophages, fibroblasts, endothelium and lung adenocarcinoma. That toolkit acquires a distinct disease role across idiopathic pulmonary fibrosis, chronic obstructive pulmonary disease, asthma, acute respiratory distress syndrome, tuberculosis, severe acute respiratory syndrome coronavirus two infection and lung cancer, generating a matrix of cell-type-by-disease configurations that no prior review has systematized. Four target axes, namely, tether occupancy, lipid flux, redox coupling and cell-type-precise delivery, converge geometrically at the interface and motivate combination strategies rather than pan-mitochondrial single-agent approaches. Inhaled lipid nanoparticles, mucus-penetrating carriers and engineered extracellular vesicles bring these targets within reach with cell-type precision. Endothelial contact-site biology is identified as the largest evidence gap and discussed candidly. The framework is offered as a roadmap for tether-axis compound development, contact-site engagement biomarkers and dual-axis inhaled therapeutics.

## 1 Introduction

Lipid droplets were long treated as inert depots. Three decades of cell biology have replaced that view with one of a dynamic organelle whose contacts with mitochondria can now be measured directly. Functional segregation experiments first showed that lipid-droplet–tethered mitochondria are biochemically distinguishable from the cytoplasmic pool, with enhanced pyruvate-driven respiration and a discrete bioenergetic role in fat storage (Benador et al., 2018). Subsequent work in liver established that peridroplet mitochondria carry higher pACC2, MFN2, and CPT1 activity than their cytoplasmic counterparts, anchoring the functional-segregation concept *in vivo* (Talari et al., 2023). Distinct dynamic regimes have been recognised at this interface. Lipid droplets and mitochondria establish a kiss-and-run pattern in cells with high lipolytic flux and an anchored, stable pattern in oxidative tissues, with the anchored configuration becoming more prevalent during brown and beige adipocyte differentiation (Cui and Liu, 2020). Proximity-proteomic dissection has since converted these observations into a defined molecular interface. An ESYT1/ESYT2–VAPB tripartite assembly was mapped at the lipid-droplet–mitochondria–endoplasmic reticulum junction, and its disruption reduced droplet-derived fatty-acid oxidation and altered tricarboxylic-acid intermediates in cells and mice (Bezawork-Geleta et al., 2025). A separate proximity-labelling effort in muscle-derived cells identified an interaction between PLIN5 and the long-chain acyl-CoA synthetase FATP4 that drives fatty-acid channelling from droplet to mitochondrion during starvation (Miner et al., 2023). A complementary line of work identified PLIN5 phosphorylation at serine 155 as a switch-like, quantitative regulator of contact-site abundance and lipid flux through the interface (Kang et al., 2026; Kien et al., 2022; Zhang et al., 2022; Griseti et al., 2024), consistent with parallel post-translational regulators of droplet–mitochondria coupling (Niu et al., 2025). The accompanying News and Views framed PLIN5-S155 as an early-stage indicator of contact-site dysfunction in metabolic-dysfunction-associated steatotic liver disease (Segalé et al., 2026). A connected line of work in adipocytes had earlier defined MIGA2 as a triple-organelle anchor that ties mitochondria to the endoplasmic reticulum and to lipid droplets and is required for efficient lipid storage, providing an additional anatomical scaffold for the contact-site concept (Freyre et al., 2019; Bórquez et al.; Peng et al., 2026). Together these advances have moved the lipid-droplet–mitochondria contact site (LDMC) from descriptive ultrastructure into the territory of mechanistically tractable, pharmacologically addressable biology (Klein et al., 2021; Herker et al., 2021; Wu et al., 2022; Zadoorian et al., 2023), with recent mechanistic refinements consolidating this view (Zhang et al., 2024; Smolk et al., 2025).

The case for treating LDMC as a respiratory-disease target rests on the lung’s unusual lipid economy. The alveolar surface depends on a continuous supply of saturated phospholipid for surfactant, manufactured by alveolar type 2 cells and packaged in lamellar bodies, lysosome-related organelles that are distinct from cytoplasmic lipid droplets but whose biogenesis is coupled to mitochondrial metabolism (Xu et al., 2023; Cañadas et al., 2020; Pranty et al., 2026). Surfactant homeostasis is also coupled to mitochondrial function. GCN5L1, a mitochondrial acetyltransferase regulator, controls lamellar-body biogenesis and trafficking in alveolar epithelial cells, linking mitochondrial acetyl-coenzyme A flux to surfactant assembly (Zhu Y. et al., 2024; Dietl and Frick, 2021; Li et al., 2022). Surfactant is reclaimed and recycled by alveolar macrophages, and dysregulation of this loop produces foam-cell–dominated injury patterns seen in chronic obstructive pulmonary disease, tuberculosis, and silicosis (Angeles-Lopez et al., 2025; Xie et al., 2025; Agarwal et al., 2021; Aloe et al., 2022). Activated lung fibroblasts add a second demand. Collagen deposition imposes a sustained requirement for mitochondrial acetyl-coenzyme A and obligates β-oxidation, and the lung progenitor plasticity needed for aberrant repair in idiopathic pulmonary fibrosis depends on fatty-acid oxidation that is itself sensitive to droplet–mitochondria flux (Han et al., 2023; Bueno et al., 2020; Tian et al., 2023). Lung adenocarcinoma adds a third demand. Spatial mapping of mitochondrial networks in non–small cell lung cancer revealed that tumours with high oxidative phosphorylation organise peridroplet mitochondrial compartments that surround and contact lipid droplets, supplying a tumour-specific bioenergetic niche, while tumours with low oxidative phosphorylation displace the network into a perinuclear configuration with remodelled cristae (Fan and Tan, 2024; Jin and Yuan, 2020; Liu et al., 2023). Each of these lung-specific pressures recruits the same molecular machinery into cell-type-specific configurations, and each invites cell-type-resolved therapeutic targeting.

The current review landscape leaves this niche unfilled. Reviews of the LDMC interface synthesise data from liver, muscle, and adipose (Fan and Tan, 2024; Wang G. et al., 2025; Enkler and Spang, 2024), without organising them around lung cell types (Herker et al., 2021; Zhang et al., 2024; Smolk et al., 2025). Reviews of lung mitochondria focus on bioenergetic dysfunction in chronic lung disease but rarely integrate contact-site mechanisms with the tether catalogue (Cloonan et al., 2020; Pokharel et al., 2024). Reviews of lung lipid pathology cover foam-cell biology and surfactant homeostasis without engaging the tether and flux machinery in mechanistic detail (Agudelo et al., 2020; Najt et al., 2020). The present synthesis is built around the proposition that the LDMC interface, when read across alveolar type 2 cells, alveolar and interstitial macrophages, fibroblasts, endothelium, and lung adenocarcinoma cells, becomes a coherent, spatial-pharmacology target class in respiratory disease, with the caveat that most lung evidence remains indirect. The contact site is approachable by inhaled lipid nanoparticles and engineered extracellular vesicles, which together open a route to cell-type-precise intervention. The following argument first establishes the LDMC molecular toolkit and its recruiting pulmonary lipid economy. It then applies this toolkit across principal lung cell types. These include alveolar type 2 cells, alveolar and interstitial macrophages, fibroblasts, the pulmonary endothelium, and lung adenocarcinoma cells. This step distinguishes solid mechanisms from provisional ones. The resulting insights converge into a disease matrix. This matrix links idiopathic pulmonary fibrosis, chronic obstructive pulmonary disease, asthma, silicosis, acute respiratory distress syndrome, tuberculosis, severe acute respiratory syndrome coronavirus two infection, and lung adenocarcinoma to specific LDMC failure modes and cell-type entry points. Throughout, the endothelial layer is acknowledged as the most poorly resolved compartment. The discussion is deliberately structured to make this gap visible rather than concealed.

## 2 The LDMC interface across the lung

### 2.1 The molecular toolkit and the pulmonary lipid economy

#### 2.1.1 A layered molecular interface

The LDMC interface is built from a small but structurally distinguishable set of perilipins, mitochondrial-outer-membrane anchors, endoplasmic-reticulum–coupled tethers, glycolytic adaptors, and lipid-transfer components. This toolkit, catalogued in liver, skeletal muscle, and adipose, can be read as a layered architecture rather than a list. Three structural classes can be defined. The first comprises droplet-side perilipins and their phosphorylation-regulated interactors. The second comprises mitochondrial-outer-membrane and endoplasmic-reticulum–anchored tethers that establish the physical contact. The third comprises adaptors and lipid-transfer modules that link contact-site occupancy to substrate flux. This organisation matters for the cell-type analyses below, because each lung cell type recruits these classes in characteristic combinations.

Perilipin-family proteins furnish the LDMC’s droplet-side interface. PLIN5 is the most extensively dissected member. Beyond its scaffolding of adipose triglyceride lipase (ATGL) and its co-activator CGI-58 at the droplet surface, PLIN5 docks long-chain fatty acids onto the long-chain acyl-coenzyme A synthetase FATP4 at droplet–mitochondria junctions, channelling fatty acids directly from droplet to mitochondrion during starvation rather than allowing them to enter the bulk cytoplasmic pool (Miner et al., 2023; Kien et al., 2022; Smolk et al., 2025). A separate function for PLIN5 has been resolved in muscle, where lipid-droplet–derived monounsaturated fatty acids traffic via PLIN5 to the nuclear deacetylase SIRT1 and allosterically activate it, coupling mobilised acyl chains to a downstream transcriptional programme (Zhang et al., 2022; Griseti et al., 2024; Najt et al., 2020). Quantitative control of contact-site abundance is exerted by phosphorylation. The phosphomimetic PLIN5-S155E variant collapses droplet–mitochondria coupling, whereas the phospho-deficient S155A variant amplifies it, expanding droplets and reducing lipotoxicity under a Western diet (Kang et al., 2026; Zhang et al., 2022; Niu et al., 2025). PLIN2 covers droplets in cells that do not strongly express PLIN5, including most non-oxidative epithelial cells, and is recruited to the LDMC by phosphorylation events that engage the glycolytic adaptor described below (Griseti et al., 2024; Zadoorian et al., 2023; Wang et al., 2020; Stephens and Johnson, 2025).

A second class of LDMC components is built from mitochondrial-outer-membrane and endoplasmic-reticulum–anchored tethers. Mitofusin 2 (MFN2) acts both as a fusion-promoting GTPase and as a heterotypic tether that, in adipose tissue, binds the lipid-droplet–localised heat-shock cognate Hsc70 to position mitochondria adjacent to droplets (Bórquez et al.; Fan and Tan, 2024). MIGA2, also known as mitoguardin-2, is anchored in the outer mitochondrial membrane and links mitochondria simultaneously to lipid droplets and to the endoplasmic reticulum, providing a triple-organelle scaffold that is required for efficient neutral-lipid storage (Freyre et al., 2019; Peng et al., 2026). A structural study has further resolved how the cytoplasmic lipid-transfer domain of MIGA2 binds phosphatidic acid and phosphatidylglycerol and reaches across the contact interface to support inter-organelle lipid transport at endoplasmic-reticulum–mitochondrion contact sites (Kim et al., 2022). A complementary endoplasmic-reticulum–bridged tether is provided by the extended synaptotagmins ESYT1 and ESYT2, which assemble with VAPB at a lipid-droplet–mitochondria–endoplasmic-reticulum tripartite junction whose disruption reduces droplet-derived fatty-acid oxidation, depletes tricarboxylic-acid intermediates, and causes lipotoxic stress in cells and in Esyt1/Esyt2-deficient mice (Bezawork-Geleta et al., 2025). Trafficking factors expand this layer. SNAP23 has been implicated in coupling droplet–mitochondria fusion-like events to fatty-acid mobilisation, and Rab8a, after phosphorylation by AMP-activated protein kinase, binds PLIN5 to recruit ATGL and accelerate droplet-to-mitochondrion fatty-acid flow, an axis catalogued across recent integrative reviews (Fan and Tan, 2024; Wang G. et al., 2025).

The third class translates contact-site occupancy into substrate flux. The glycolytic enzyme phosphofructokinase-liver (PFKL) phosphorylates PLIN2 under energetic stress, enabling PLIN2 to recruit the rate-limiting fatty-acid-import enzyme carnitine palmitoyltransferase 1A (CPT1A) to the droplet–mitochondria interface, thereby tightening tethering and amplifying β-oxidation. This mechanism was established in hepatocellular carcinoma, where PFKL- and PLIN2-phosphorylation levels track poor prognosis; it has not yet been demonstrated in lung tissue (Zhang et al., 2024; Smolk et al., 2025; Meng et al., 2024). This PFKL–PLIN2–CPT1A axis ties cytosolic glycolytic flux directly to droplet-derived oxidative output through the contact site and represents one of the most pharmacologically addressable elements of the LDMC toolkit. Lipid-transfer is also assisted at the droplet side by acyl-coenzyme A synthetase long-chain family members, which channel activated acyl chains into mitochondrial CPT1A loading.

**FIGURE 1:**
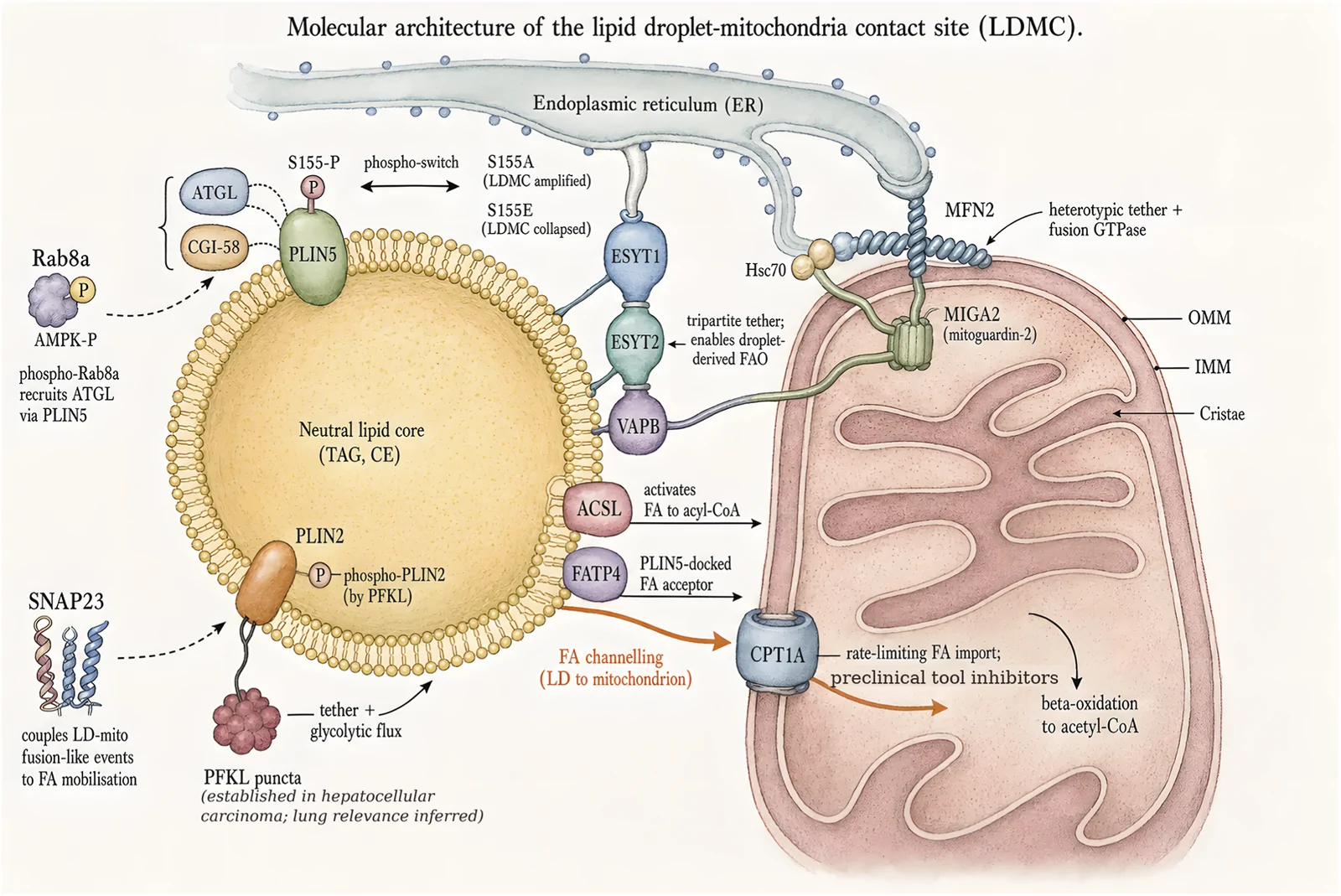
Layered molecular architecture of the lipid droplet–mitochondria contact (LDMC). Layered molecular architecture of the lipid droplet–mitochondria contact (LDMC). Three structural classes build the LDMC interface. a, Droplet-side perilipins. PLIN5 scaffolds ATGL and CGI-58 and docks fatty acids onto FATP4 at the droplet–mitochondria junction; the PLIN5-Ser155 phospho-switch sets contact-site abundance, with S155A (phospho-deficient) amplifying droplet–mitochondria coupling and S155E (phosphomimetic) collapsing the contact. PLIN2 covers droplets in non-oxidative cells and is recruited by phosphorylation events feeding into **(C)**. b, MOM- and ER-anchored tethers. MFN2 tethers mitochondria to droplet-localised Hsc70. MIGA2 anchors in the outer mitochondrial membrane and simultaneously engages droplet and endoplasmic reticulum as a triple-organelle scaffold. ESYT1/2 assemble with VAPB at a droplet–mitochondria–ER tripartite junction. SNAP23 and AMPK-phosphorylated Rab8a extend the tether layer. c, Substrate-flux class. PFKL phosphorylates PLIN2, licensing PLIN2 to recruit CPT1A to the contact and amplifying β-oxidation (mechanism established in hepatocellular carcinoma; lung relevance inferred); ACSLs channel acyl chains to CPT1A. Node shading marks druggability status: green, inhibitors in clinical use for other indications; amber, preclinical tool compounds or activators in development; grey, no direct ligand yet.

Imaging now resolves the LDMC at near-molecular precision. Cryogenic correlative light and electron microscopy of alveolar type 2 cells has reconstructed lamellar-body membrane architecture and its juxtaposition with mitochondria (Klein et al., 2021). Because lamellar bodies are lysosome-related organelles rather than lipid droplets, this juxtaposition templates a distinct lamellar-body–mitochondria interaction, not an LDMC in the strict sense (Section 2.1.2). Live-cell and super-resolution approaches such as lattice light-sheet microscopy capture the kiss-and-run versus anchored dynamic regimes at physiologically relevant time scales, and focused-ion-beam scanning electron microscopy resolves the three-dimensional anchor geometry. Together, these tools permit quantitative read-outs of contact-site abundance and dynamics that could, in principle, translate into pharmacological end points. The druggability of individual LDMC components varies widely and remains largely prospective. PLIN5-S155 phosphorylation is reversible and downstream of established kinases, making it a candidate for indirect pharmacological control; MFN2 has small-molecule activators in preclinical development; MIGA2 lipid binding may be tractable through fragment-based screening once a structural compound library becomes available; and CPT1A is targeted by preclinical tool inhibitors such as etomoxir, whose dose-dependent off-target effects preclude clinical use (Section 3.1). The layered LDMC architecture and its druggable nodes are summarised as a molecular schematic in Figure 1 and catalogued component-by-component in Table 1. Cell-type-specific tether stoichiometries in alveolar type 2 cells, alveolar and interstitial macrophages, lung fibroblasts, and pulmonary endothelium have not been systematised at the level of detail now available for liver and skeletal muscle, and this gap is engaged below.

**TABLE 1:** The LDMC molecular toolkit: twelve components across three structural classes.

Component	Structural class	Molecular function	Lung-relevant role	Druggability status	Key references
PLIN5	Droplet-side perilipin	Scaffolds ATGL and CGI-58 on the droplet; docks long-chain fatty acids onto FATP4 at LD–mitochondria junctions to channel them directly from droplet to mitochondrion during starvation. In muscle, also traffics monounsaturated fatty acids to nuclear SIRT1 for allosteric activation	Positions AT2 and lung oxidative cells for peridroplet FA channelling; candidate governor of myofibroblast lipid mobilisation; expected but unquantified regulator of PMVEC LDMC flux	No direct ligand yet; indirect control via phosphorylation kinases; S155 phospho-antibody as biomarker candidate	Miner et al. (2023), Kien et al. (2022), Zhang et al. (2022), Griseti et al. (2024), Smolk et al. (2025), Wang et al. (2020)
PLIN5-S155 phospho-switch	Droplet-side regulatory node	Ser155 phosphorylation sets LD–mitochondria contact abundance: S155A (phospho-deficient) amplifies coupling, expands droplets and reduces lipotoxicity; S155E (phosphomimetic) collapses the contact	Untested but tractable in AT2 cells under mechanical strain/hyperoxia and in fibroblast reparative vs. pathological state transitions; candidate master switch	Reversible post-translational switch downstream of established kinases; S155 phospho-antibody proposed for transbronchial biopsy and BAL biomarker use	Kang et al. (2026), Zhang et al. (2022), Niu et al. (2025)
PLIN2 (ADRP)	Droplet-side perilipin	Dominant droplet coat in cells with low PLIN5. Recruited to LDMC by PFKL-mediated phosphorylation, enabling interaction with CPT1A at the contact	Prognostic biomarker in LUAD (high PLIN2 → poor differentiation, vascular/pleural invasion, shorter recurrence-free survival). PLIN2 KO reduces LD accumulation, proliferation and migration in A549/H1299	CRISPR-tractable in LUAD lines; no small-molecule inhibitor yet; PFKL kinase axis provides indirect entry	Griseti et al. (2024), Zadoorian et al. (2023), Stephens and Johnson (2025), Kim et al. (2022), Adamcakova and Mokra (2021)
MFN2 (Mitofusin-2)	Tether (OMM-anchored)	OMM GTPase acting as both a homotypic mitochondrial-fusion factor and a heterotypic LD–mitochondrion tether that binds droplet-localised Hsc70	Conditional AT2 deletion of MFN1/MFN2 causes morbidity, spontaneous lung fibrosis and disabled surfactant phospholipid and cholesterol synthesis	Small-molecule mitofusin activators in development; not yet tested in respiratory disease	Bórquez et al. (2024), Fan and Tan (2024)
MIGA2 (Mitoguardin-2)	Tether (OMM-anchored, triple-organelle)	OMM anchor engaging droplet and endoplasmic reticulum simultaneously; provides triple-organelle scaffold required for efficient neutral-lipid storage; cytoplasmic lipid-transfer domain binds phosphatidic acid and phosphatidylglycerol	Candidate scaffold at collagen-secretion hotspots in myofibroblasts; untested in AT2 and PMVEC.	Lipid-binding pocket structurally resolved; fragment-based screening tractable; no clinical ligand yet.	Freyre et al. (2019), Peng et al. (2026), Meng et al. (2024))
ESYT1/2–VAPB triad	Tether (ER-anchored, tripartite)	Extended synaptotagmins ESYT1 and ESYT2 assemble with ER-resident VAPB at an LD–mitochondria–ER tripartite junction; disruption reduces droplet-derived β-oxidation and depletes TCA intermediates	Anatomical template for LDMC in AT2 (cryo-CLEM); testable in fibroblasts as the mechanical-load-to-lipid-flux interface	No direct ligand; VAPB and ESYT proximity assays available for target-engagement read-outs	Bezawork-Geleta et al. (2025)
SNAP23	Flux adaptor/trafficking	SNARE-family protein coupling LD–mitochondrion fusion-like events to fatty-acid mobilisation	Unprofiled in lung; plausibly relevant to alveolar macrophage surfactant-recycling loop	Genetic tractability only; no direct pharmacological modulator	Fan and Tan (2024)
Rab8a	Flux adaptor/trafficking	Small GTPase that, after AMPK phosphorylation, binds PLIN5 to recruit ATGL and accelerate droplet-to-mitochondrion FA flow	AMPK-coupled entry point for LDMC control in energetically stressed AT2 and macrophages; not directly assayed	Indirect control via AMPK activators (metformin class); no direct Rab8a ligand	Fan and Tan (2024), Enkler and Spang (2024)
FATP4 (SLC27A4)	Flux adaptor/lipid transfer	Long-chain acyl-CoA synthetase that accepts PLIN5-docked fatty acids at LD–mitochondria junctions and activates them for mitochondrial import	Candidate catalytic node for AT2 and myofibroblast peridroplet FA channelling; untested in PMVEC.	Selective FATP4 inhibitors reported preclinically (dermatology); repurposable with appropriate pulmonary delivery	Miner et al. (2023), Kien et al. (2022), Smolk et al. (2025))
ACSL family	Flux adaptor/lipid transfer	Activates fatty acids as acyl-CoA at the droplet side, channelling acyl chains into CPT1A loading and assisting lipid transfer across the contact	ACSL4 downregulated by CPT1A–c-Myc–NRF2/GPX4 in LUAD cancer stem cells, linking LDMC to ferroptosis resistance and immune-checkpoint response	ACSL4 inhibitors (rosiglitazone-derived class) preclinically active; ferroptosis-modulator programmes ongoing	Fan et al. (2023), Xu et al. (2025)
PFKL–PLIN2–CPT1A axis	Flux adaptor/bifunctional tether axis	Under energetic stress, PFKL phosphorylates PLIN2, enabling recruitment of CPT1A to the LD–mitochondria interface; tightens tethering and amplifies β-oxidation while directing glycolytic flux to peridroplet oxidation	The single most experimentally complete LDMC mechanism in a disease context; central regulator in LUAD; explains limited glycolysis-only therapy efficacy	PFKL small-molecule inhibitors in preclinical development; CPT1A targeted by preclinical tool inhibitors (etomoxir; not clinically accessible owing to off-target toxicity); eight-gene LD–Mito risk score validated in TCGA. Axis mechanism established in hepatocellular carcinoma; lung relevance inferred	Zhang et al. (2024), Smolk et al. (2025), Adamcakova and Mokra (2021)
CPT1A	Flux adaptor (catalytic node)	OMM carnitine palmitoyltransferase 1A, rate-limiting for mitochondrial FA import; recruited to LD–mitochondria contacts by phospho-PLIN2; in macrophages, also binds the BH3 domain of mitochondrial Bcl-2	Bidirectional: overexpression alleviates LPS-induced acute lung injury (protective in AT2/epithelium in ARDS); sustains apoptosis resistance in lipid-laden macrophages in IPF and COPD.	Preclinical tool inhibitors (etomoxir class; no approved clinical CPT1A inhibitor); CPT1A–Bcl-2 interface offers macrophage-selective anti-fibrotic entry; combinable with immune-checkpoint blockade	Zhang et al. (2024), Smolk et al. (2025), Adamcakova and Mokra (2021)

Because the evidence base behind this toolkit is uneven, we grade every mechanism discussed in this review into three tiers and signal the tier wherever a claim is made. Tier (i) comprises direct lung LDMC evidence: structural or functional data that resolve lipid-droplet–mitochondria contacts in lung cells or tissue, currently limited to spatial-proteomic mapping of peridroplet mitochondria in non–small cell lung cancer (Han et al., 2023; Jin and Yuan, 2020; Liu et al., 2023). Cryogenic correlative light and electron microscopy of lamellar-body–mitochondria juxtaposition in alveolar type 2 cells (Klein et al., 2021) is not classified as Tier (i) LDMC evidence, because lamellar bodies are lysosome-related organelles rather than cytoplasmic lipid droplets; we treat that juxtaposition as a distinct organelle interaction and grade it as Tier (ii) (Section 2.1.2). Tier (ii) comprises indirect lung evidence: metabolic, transcriptomic, genetic or pharmacological data from lung systems that implicate LDMC components without resolving the contact site itself; the majority of the lung literature, including the MFN1/MFN2, HMGCS2, CPT1A–Bcl-2 and GCN5L1 axes, sits here. Tier (iii) comprises cross-tissue analogy: mechanisms established in liver, muscle or adipose including the PLIN5-S155 phospho-switch, MIGA2 triple-organelle anchoring, ESYT1/2–VAPB tripartite tethering and the PFKL–PLIN2–CPT1A axis, whose operation in lung cells is inferred rather than demonstrated. This grading is carried through the text, Tables 2, 3 and the figures.

**TABLE 2:** LDMC configurations across six lung cell types.

Cell type	Dominant LDMC configuration	Key molecular components	Physiological role	Pathological rewiring	Evidence strength/gap	Key references
Alveolar type 2 (AT2)	Lamellar-body–coupled peridroplet architecture; MFN1/MFN2 obligate for surfactant lipid handling; mitochondrial acetyl-CoA flux to lamellar bodies regulated by GCN5L1; cryo-CLEM resolves lamellar-body–mitochondria juxtaposition, a distinct organelle interaction (lamellar bodies are lysosome-related, not lipid droplets) that is not classified as LDMC evidence Surfactant-recycling LDMC with lysosomal–mitochondrial coordination; foam-cell-permissive under chronic lipid input; CPT1A docks on mitochondrial Bcl-2 via its BH3 domain to suppress apoptosis	MFN1/MFN2; HMGCS2; FASN; GCN5L1; PLIN5 and PLIN2 (expected); ESYT1/2–VAPB (anatomically templated); MIGA2 (candidate)	Synthesises saturated phosphatidylcholines (DPPC) via the CDP-choline pathway, packages them into lamellar bodies, secretes surfactant and mediates alveolar repair	In IPF, AT2 mitochondrial permeability transition pore opening propagates injury; MFN1/MFN2 loss drives spontaneous fibrosis and disables surfactant lipid synthesis; HMGCS2 downregulation releases lysophosphatidylcholine that activates fibroblasts; FASN deficiency exacerbates bleomycin fibrosis	[Indirect lung evidence; the lamellar-body–mitochondria juxtaposition is a distinct organelle interaction, not LDMC] Mechanistically deep for MFN1/MFN2, HMGCS2 and FASN axes; single-cell PLIN5/PLIN2/ESYT1/2/MIGA2 stoichiometry not quantified; PLIN5-S155 lung-specific mapping open	Hao et al. (2024), Chung et al. (2019), Sever et al. (2021), Sun et al. (2024), Yang et al. (2024), Chen et al. (2023a), Kallinos et al. (2025)
Alveolar macrophage	Surfactant-recycling LDMC with lysosomal–mitochondrial coordination; foam-cell-permissive under chronic lipid input; CPT1A docks on mitochondrial Bcl-2 via its BH3 domain to suppress apoptosis	PLIN2 (foam-cell coat); CPT1A; mitochondrial Bcl-2; CD44 (surfactant scavenging); mitochondrial calcium uniporter; FABP5 (macrophage subsets)	Reclaims and recycles surfactant from the alveolar lumen; transient foam phenotype under physiology; CD44 loss disrupts surfactant clearance	In COPD, cholesterol overload drives mitochondrial dysfunction, ROS and pro-inflammatory signalling; in IPF, CPT1A–Bcl-2 disruption targets apoptosis-resistant lipid-laden macrophages; in TB and silicosis, similar foam-cell states dominate	[Indirect lung evidence; functional and metabolic lung data without contact-resolving LDMC measurement]Multiple axes with cross-sectional and mouse-genetic support (CD44, CPT1A–Bcl-2, cholesterol-overload multi-omics); PFKL–PLIN2–CPT1A axis in lung macrophages not yet resolved	Agudelo et al. (2020), Najt et al. (2020), Lee et al. (2024a), Morán-Lalangui et al. (2025), Little et al. (2025), Scur et al. (2022), Hernández-Hernández et al. (2024), Stevenson et al. (2023), Giambelluca et al. (2025), Ding et al. (2024), Knight et al. (2018)
Interstitial macrophage	LDMC inferred by analogy but not experimentally profiled in human respiratory disease; predominantly monocyte-derived under inflammation; surveys perivascular and peribronchial lipid pools	Expected: PLIN2, PLIN3 (TAM context), CPT1A, FABP5; direct measurement absent	Parenchymal lipid surveillance and interstitial inflammatory homeostasis; likely bridge between endothelial lipid transit and the alveolar surfactant loop	Not directly profiled in IPF, COPD or asthma cohorts despite scRNA-seq of a distinct lipid-metabolic profile; PLIN3 recruits M2 TAMs in LUAD, establishing a paracrine tumour axis	[Cross-tissue analogy] Under-systematised; tether and flux machinery unprofiled in any chronic human lung disease. Highest-yield systematisation opportunity in the macrophage compartment	Meng et al. (2024), Adamcakova and Mokra (2021), Lung et al. (2022)
Fibroblast/myofibroblast	Substrate-hungry, matrix-secreting LDMC with sustained acetyl-CoA sink for collagen synthesis; peridroplet mitochondrial coupling not yet imaged at high resolution in primary lung fibroblasts	Expected but not directly quantified: PLIN2, PLIN5 (candidate), MIGA2 (candidate at collagen-secretion hotspots), ESYT1/2–VAPB (candidate at mechanical-load interface), PFKL puncta	Healthy lung: quiescent stromal cells with modest lipid reserves and low LDMC demand. After injury: sustained matrix production at high energetic and biosynthetic cost	In IPF and scleroderma-associated ILD, myofibroblasts show LDMC-permissive metabolomic signatures with elevated LD-associated transcripts and altered β-oxidation intermediates; LPA drives pro-fibrotic signalling	[Indirect metabolic evidence] Motivated by scRNA-seq, mini-review and cross-fibrotic metabolomics; contact site IMAGING-TRACTABLE, NOT YET IMAGED; PLIN5-S155 directionality unresolved	Li et al. (2025b), Geng et al. (2021), Kumari et al. (2025), Lee et al. (2022), Wang et al. (2025b), Zhang and Michelakis (2021)
Pulmonary microvascular endothelium (PMVEC)	Largest evidence gap in the field. LDMC configuration in the pulmonary capillary bed has not been experimentally resolved by methods available for AT2/macrophages/fibroblasts	Candidate inferred set only: PLIN5-S155 phospho-switch, MIGA2, ESYT1/2–VAPB, PFKL–PLIN2 (inflammatory-milieu tether); direct tether stoichiometry not measured	Fatty-acid β-oxidation-dependent for membrane phospholipid biosynthesis during proliferation and barrier maintenance; obligate first-pass site for postprandial chylomicron remnants	In ARDS, endothelial mitochondrial dysfunction induced by LPS or hyper-inflammatory patient plasma is reversed by MSC-derived mitochondrial transplantation, restoring respiration and barrier function	[Cross-tissue analogy] Field’s largest gap. Minimum first experimental package: live-cell imaging of LD–mitochondrion contact dynamics in primary human PMVEC under inflammation/hypoxia, paired with PLIN5-S155 and MIGA2 read-outs	Li et al. (2025b), Wang et al. (2025b), Zhang and Michelakis (2021), Lee et al. (2024b), Wang et al. (2024a), McClintock et al. (2022)
Lung adenocarcinoma (LUAD)	The only lung compartment with direct LDMC evidence: spatial proteomics and electron tomography resolve dense LD–mitochondrion contacts and OXPHOS-leaning peridroplet mitochondria in high-grade LUAD (Han et al., 2023; Jin and Yuan, 2020; Liu et al., 2023). The PFKL puncta that act as bifunctional tethers were defined in hepatocellular carcinoma (Adamcakova and Mokra, 2021) and are inferred, not demonstrated, in LUAD.	PFKL (bifunctional glycolytic + tether); PLIN2 (recruited by PFKL phosphorylation); CPT1A (recruited by phospho-PLIN2); PLIN3 (M2 TAM interface); ATGL; ACSL4 (downregulated); miR-365-3p (suppressed)	Non-transformed lung epithelium: normal FA channelling. In LUAD: co-opted into a tumour-specific bioenergetic niche with peridroplet mitochondria contacting PLIN2-decorated droplets	High PLIN2 associates with poor differentiation, invasion, advanced stage and shorter recurrence-free survival; PLIN2 KO reduces LD accumulation, proliferation and migration; CPT1A sustains ferroptosis resistance and drives immune-checkpoint response	[Direct LDMC evidence (spatial proteomics/electron tomography; 31,35,36) + cross-tissue analogy (PFKL tether; 47)] Demonstrated in LUAD: PLIN2 prognostic biomarker, PLIN3–TAM axis, CPT1A–ferroptosis resistance, eight-gene LD–Mito risk score, and spatial-proteomic mapping of peridroplet mitochondria. Inferred from hepatocellular carcinoma: the PFKL–PLIN2–CPT1A tether-and-flux mechanism	Jin and Yuan (2020), Adamcakova and Mokra (2021), Fan et al. (2023), Torresano et al. (2022), Yang et al. (2025), Xu et al. (2025), Eltayeb et al. (2022)

**TABLE 3:** Disease matrix of LDMC failure modes and therapeutic routes across eight respiratory illnesses.

Disease	Dominant failure mode	Cell-type entry point	LDMC axis involved	Direction of change	Therapeutic route	Key references
Idiopathic pulmonary fibrosis (IPF)	Distributed failure across alveolar, mesenchymal and macrophage compartments: AT2 mitochondrial dysfunction and AT2-derived lipid signalling to fibroblasts, combined with an apoptosis-resistant lipid-laden macrophage compartment	AT2 (mitochondrial protection); lung macrophage (CPT1A–Bcl-2 axis); myofibroblast (imaging-tractable, not yet imaged)	MFN1/MFN2-mediated AT2 lipid handling; HMGCS2–LPC–fibroblast paracrine axis; MPTP; CPT1A–Bcl-2 interaction (CPT1A binds mitochondrial Bcl-2 BH3 domain to block macrophage apoptosis)	MFN1/2 ↓; HMGCS2 ↓; mitochondrial Ca uniporter ↑; mitochondrial Bcl-2 ↑; CPT1A–Bcl-2 ↑ (apoptosis resistance); fibroblast FAO/glycerolipid biosynthesis/cholesterol handling ↑	Combination repurposing: (i) CPT1A–Bcl-2 disruption in macrophages + AT2 mitochondrial protection; (ii) LPAR1 antagonism + LDMC modulators; (iii) mucus-penetrating inhalable LNP delivery of Cyb5R3/BMP4 mRNAs; (iv) nintedanib/pirfenidone backbone	Hao et al. (2024), Chen et al. (2023a), Gu et al. (2022), Bonella et al. (2023), Wang et al. (2024a), Ni et al. (2026)
Chronic obstructive pulmonary disease (COPD)	Cigarette-smoke-driven oxidative stress with cholesterol-driven macrophage mitochondrial dysfunction; ferroptosis of membrane phospholipids as unifying mediator amplified by DRP1-driven mitochondrial fission	Alveolar macrophage (dominant); AT2 cell (secondary)	Cholesterol-overload macrophage LDMC; PUFA phospholipid peroxidation (ferroptosis); DRP1-driven mitochondrial fission at LDMC.	Cholesterol handling ↑; macrophage mitochondrial function ↓; ROS ↑; ferroptosis-sensitising lipid peroxidation ↑	Small-molecule LDMC modulation: LXR-axis or statin repurposing to relieve cholesterol overload; ferroptosis stabilisation upstream of DRP1 fission; mitochondria-targeted antioxidants via inhaled LNPs	Zhu et al. (2024a), Xie et al. (2025), Guerrini et al. (2020), Giambelluca et al. (2025), Manevski et al. (2020), Fairley et al. (2023)
Asthma	Adaptive-immune-driven dysregulated lipid trafficking rather than impaired bioenergetics: myeloid FABP5 loss releases free oleate and reprograms macrophages toward M2 polarisation	Alveolar and interstitial macrophage	FABP5 checkpoint on free FA loading at LDMC; oleate-driven PPARγ signalling; upregulated β-oxidation flux through CPT1A	FABP5 ↓; free oleate ↑; macrophage FAO/TCA cycle/OXPHOS ↑; M2 polarisation ↑	Small-molecule LDMC modulation: macrophage-selective CPT1A restraint that spares antiviral/antibacterial defence; combination with inhaled corticosteroids	Lu et al. (2025), Wu et al. (2026), Huang et al. (2022), Esteves et al. (2021)
Silicosis/pneumoconiosis	Recurrent alveolar-macrophage lipid uptake without effective resolution, driven by chronic exogenous mineral dust; sustained foam-cell state convergent with COPD/TB phenotypes	Alveolar macrophage	Foam-cell LDMC remodelling secondary to persistent mineral-dust-driven lipid uptake; surfactant scavenging (CD44) overwhelmed	Macrophage lipid uptake ↑ (persistent); surfactant clearance ↓; foam-cell state persistent	Small-molecule LDMC stabilisation: shift mineral-laden macrophages toward pro-resolution state; early intervention in occupational cohorts to prevent fibrotic remodelling	Zhu et al. (2024a), Xie et al. (2025), Aloe et al. (2022), Li et al. (2025a), Guerrini et al. (2020)
Acute respiratory distress syndrome (ARDS)	Acute pulmonary endothelial barrier failure superimposed on alveolar epithelial injury; mitochondrial dysfunction and pulmonary oedema as principal mediators; endothelial LDMC inferred but not yet imaged	Pulmonary microvascular endothelium AND AT2 cell in parallel	Endothelial mitochondrial supply limitation (LDMC inferred); AT2 CPT1A-mediated β-oxidation as protective node against LPS injury	Endothelial mitochondrial respiration ↓; barrier integrity ↓; alveolar inflammation and apoptosis ↑; CPT1A protective when overexpressed in AT2/epithelium	Mitochondrial transplantation: MSC-derived mitochondria transferred to human PMVEC restore respiration and barrier *in vitro* and attenuate LPS injury in mice; exogenous mitochondria as delivery platform	Wang et al. (2025b), Zhang and Michelakis (2021), Lee et al. (2024b), Wang et al. (2024a), McClintock et al. (2022)
Tuberculosis (TB)	Macrophage LDMC exploited by *M. tuberculosis*: LD formation is host-driven (IFN-γ-induced, HIF-1α-dependent) for granuloma function, but direct infection counteracts the FAO alternative-activation programme	Alveolar macrophage (resists glycolytic foam-cell conversion better than BMDMs); interstitial macrophage (candidate)	IFN-γ/HIF-1α/Hig2 protective LD-formation programme; competing glycolytic foam-cell axis under direct mycobacterial infection; eicosanoid production dependent on immune context	IFN-γ/HIF-1α ↑ (protective); glycolytic reprogramming and lipid accumulation ↑ under infection (pathogenic); FAO-driven alternative activation ↓	Host-directed therapy: nudge macrophage LDMC toward IFN-γ/HIF-1α-protective configuration to shorten antibiotic-treatment duration; exploit alveolar vs. interstitial macrophage compartment window	Agarwal et al. (2021), Kalam et al. (2023), Mohareer et al. (2020), Genoula et al. (2020), Chen et al. (2024), Nardacci et al. (2021)
COVID-19 (SARS-CoV-2)	Acute viral co-option of LDMC: SARS-CoV-2 ORF3a induces microlipophagy, expands lipid-droplet reserves and remodels perinuclear membranes for replication-organelle biogenesis	AT2 (viral entry); macrophage (inflammatory relay)	ORF3a-induced microlipophagy at LDMC; SARS-CoV-2-driven remodelling of lipid metabolism, FA biosynthesis and mitochondrial function; novel droplet–membrane contact sites for replication-organelle biogenesis	Microlipophagy ↑; LD reserves diverted to replication-organelle biogenesis (rewired); mitochondrial function ↓	Small-molecule LDMC stabilisation: block viral LDMC co-option without compromising the antiviral interferon response; candidate repositioning of LDMC-engagement modulators	Dias et al. (2020), Wang et al. (2023), López-Ayllón et al. (2024), Zhang et al. (2025), Shi et al. (2024), Cevallos et al. (2025)
Lung adenocarcinoma (LUAD)	Mixed demonstrated/inferred: dense LD–mitochondria contacts and peridroplet OXPHOS are directly resolved in LUAD by spatial proteomics and electron tomography; the PFKL puncta that tether PLIN2-decorated droplets to mitochondria and recruit CPT1A are established in hepatocellular carcinoma and inferred in LUAD.	LUAD tumour cell (primary); tumour-associated M2 macrophage (secondary, via PLIN3)	PFKL–PLIN2–CPT1A tether-and-flux axis; CPT1A–c-Myc–NRF2/GPX4 ferroptosis-resistance loop with ACSL4 downregulation; PLIN3-recruited M2 TAM paracrine axis; miR-365-3p suppression of CPT1A	PFKL bifunctional activity ↑; PLIN2 ↑; CPT1A ↑ (ferroptosis resistance); ACSL4 ↓; PLIN3 ↑; miR-365-3p ↓	Combination therapy (preclinical): CPT1A inhibition (tool compounds such as etomoxir; no approved clinical inhibitor) + immune-checkpoint blockade via ferroptosis induction — the CPT1A–ferroptosis mechanism is demonstrated in LUAD models, whereas PFKL inhibitors target a tether mechanism established in hepatocellular carcinoma and inferred in LUAD; LD–Mito eight-gene risk score for patient selection	Jin and Yuan (2020), Adamcakova and Mokra (2021), Fan et al. (2023), Yang et al. (2025), Lung et al. (2022), Xu et al. (2025), Eltayeb et al. (2022)

#### 2.1.2 Three pulmonary lipid demands that recruit the toolkit

The lung imposes three lipid demands that few other organs combine in a single tissue. Surfactant phospholipid must be manufactured and secreted on a sustained basis, recycled and re-secreted by alveolar phagocytes, and metered into a tightly regulated mucociliary–alveolar interface. Each of these tracks depends on lipid-handling organelles whose biogenesis and trafficking are coupled to mitochondrial metabolism, and each is mitochondrion-coupled in ways that map onto the LDMC toolkit with varying degrees of directness.

Surfactant biogenesis begins in alveolar type 2 cells. Saturated phosphatidylcholines, most notably dipalmitoylphosphatidylcholine, are synthesised through the cytidine-diphosphate–choline pathway and packaged into lamellar bodies, lysosome-related organelles that exhibit a multilamellar architecture defined by recent cryogenic electron tomography (Klein et al., 2021; Cañadas et al., 2020; Pranty et al., 2026). Lamellar-body biogenesis is mitochondrion-coupled. The mitochondrial-acetyltransferase regulator GCN5L1 controls lamellar-body assembly and intracellular trafficking in alveolar epithelial cells, linking acetyl-coenzyme A flux to surfactant packaging (Xu et al., 2023; Pranty et al., 2026; Dietl and Frick, 2021; Li et al., 2022). Lamellar bodies are nevertheless lysosome-related organelles, biogenetically and compositionally distinct from cytoplasmic lipid droplets. The GCN5L1-dependent coupling of lamellar-body biogenesis to mitochondrial acetyl-coenzyme A flux is therefore best described as a distinct lamellar-body–mitochondria interaction rather than an LDMC in the strict sense, and throughout this review we treat it as such while noting where the two organelle systems share regulatory machinery.

After secretion into the alveolar lumen, the surfactant pool is reclaimed by alveolar macrophages and recycled. This loop demands sustained lysosomal–mitochondrial coordination, and its disruption is now recognised as a unifying axis across chronic obstructive pulmonary disease, tuberculosis, and silicosis, in which surfactant components accumulate in injury-associated macrophages and shape a foam-cell phenotype that supports persistent inflammation in COPD-, silicosis-, and TB-associated foam-cell phenotypes (Zhu Y. et al., 2024; Xie et al., 2025; Agudelo et al., 2020) and adjacent macrophage-driven inflammatory states (Agarwal et al., 2021; Aloe et al., 2022; Adamcakova and Mokra, 2021). The same loop is hijacked by viral infection. Severe acute respiratory syndrome coronavirus 2 ORF3a reprograms host lipid metabolism by inducing microlipophagy, expanding lipid-droplet reserves and remodelling perinuclear membranes to support replication-organelle biogenesis (Li Z. et al., 2025; Dias et al., 2020; Wang et al., 2023; López-Ayllón et al., 2024). These observations show that the surfactant-recycling loop is not a passive housekeeping function but a metabolically active LDMC-adjacent compartment that disease processes can rewire.

A second lung-specific demand arises from collagen-producing fibroblasts. Aberrant repair in idiopathic pulmonary fibrosis depends on lung-progenitor plasticity that is itself sustained by fatty-acid oxidation, and conditional impairment of this pathway reorganises alveolar regeneration (Angeles-Lopez et al., 2025; Bueno et al., 2020; Tian et al., 2023). Mechanistically, collagen synthesis is a sustained acetyl-coenzyme A sink, and droplet-derived fatty acids channelled through the LDMC interface help meet that demand without committing the cell to lipotoxic free-fatty-acid exposure. The third demand is supplied by tumour cells. Spatial mapping of mitochondrial networks in non–small cell lung cancer has shown that oxidative-phosphorylation-high tumours organise peridroplet mitochondrial compartments that physically contact lipid droplets and sustain a tumour-specific bioenergetic niche, whereas oxidative-phosphorylation-low tumours displace the mitochondrial network into a perinuclear configuration with remodelled cristae (Han et al., 2023; Jin and Yuan, 2020; Liu et al., 2023; Zhang et al., 2025). This finding extends the peridroplet concept beyond brown adipocytes and hepatocytes into a clinically relevant lung context.

**FIGURE 2:**
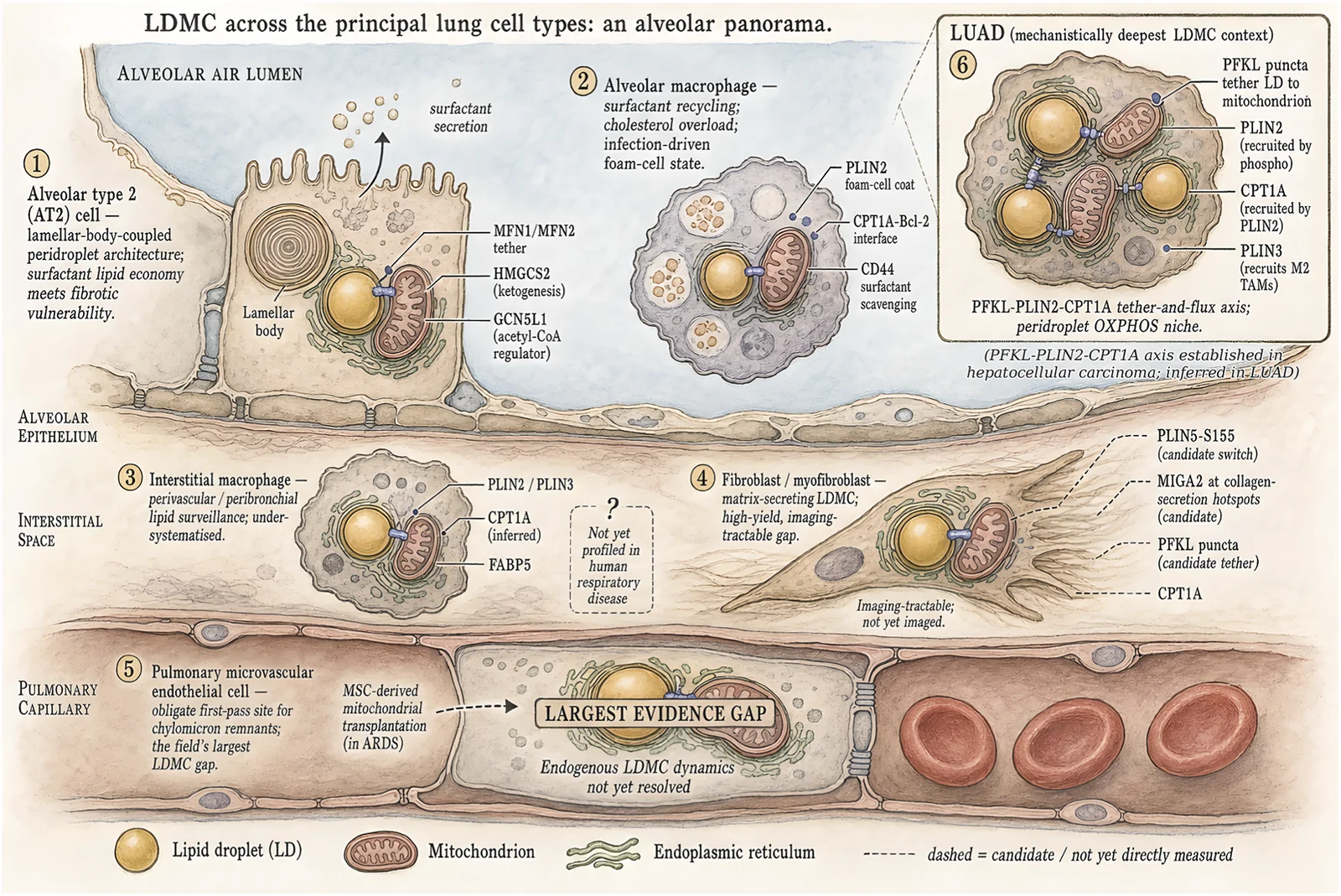
Cell-type-specific rewiring of the LDMC across the six principal lung compartments. Each panel schematises the dominant LDMC configuration, the molecular components engaged, and the direction of pathological change (↑ increased, ↓ decreased). Solid connectors denote mechanisms supported by direct LDMC evidence (contact-resolving measurement in the indicated lung cell type, currently only the LUAD spatial-proteomics dataset); dashed connectors denote mechanisms supported by indirect lung evidence or inferred by cross-tissue analogy. Lamellar-body–mitochondria coupling in panel a is a distinct organelle interaction, not an LDMC, and is shown as a dashed connector. a, Alveolar type 2 cell. Lamellar-body–coupled peridroplet architecture supported by MFN1/MFN2-obligate fusion, GCN5L1-regulated acetyl-CoA flux, HMGCS2 ketogenesis and FASN surfactant-lipid synthesis; in IPF, MFN1/MFN2↓ and HMGCS2↓ release lysophosphatidylcholine that activates fibroblasts. b, Alveolar macrophage. CD44-mediated surfactant uptake feeds a foam-cell-prone cholesterol pool; a CPT1A–Bcl-2 interaction suppresses apoptosis and yields the lipid-laden compartment characteristic of IPF and COPD. c, Interstitial macrophage. FABP5-directed handling with oleic-acid accumulation biases IL-4-driven M2 polarisation, connecting macrophage LDMC to asthma. d, Fibroblast/myofibroblast. PLIN5–FATP4-like peridroplet channelling and MIGA2 tethering (both inferred) meet a collagen-synthesis fatty-acid-oxidation demand. e, Pulmonary microvascular endothelium. The field’s largest evidence gap: chylomicron first-pass processing and ARDS-relevant mitochondrial supply, but tether stoichiometries not quantified. f, LUAD. The most mechanistically complete context: the PFKL–PLIN2–CPT1A axis (established in hepatocellular carcinoma, inferred in LUAD) is proposed to drive ferroptosis resistance, PLIN2 is a validated prognostic biomarker, and PLIN3 interfaces LUAD with M2-polarised tumour-associated macrophages.

Across these three demands, related molecular machinery is recruited into cell-type-specific configurations. In the secretory limb, lamellar-body–mitochondria coupling (a distinct organelle interaction; Section 2.1.2) organises surfactant assembly, foam-macrophage cholesterol metabolism couples LDMC remodelling to surfactant uptake and turnover, fibroblast fatty-acid oxidation meets a collagen-synthesis demand through PLIN5–FATP4-like channelling inferred from other tissues, and tumour peridroplet mitochondria reuse the anchored configuration described for brown adipocytes. The following subsections take up each cell type in turn; Figure 2 provides a cell-type-resolved overview and Table 2 summarises the dominant LDMC configuration, molecular components and evidence strength for each compartment.

### 2.2 Cell-type rewiring across the lung

#### 2.2.1 Alveolar type 2 cells: surfactant economy meets fibrotic vulnerability

Alveolar type 2 cells are the lung’s lipid-packaging specialists. They synthesise and secrete surfactant phospholipid, mediate alveolar repair, and contain lamellar bodies, lysosome-related organelles that are distinct from cytoplasmic lipid droplets but whose biogenesis is tightly coupled to mitochondrial metabolism. Mitochondrial coupling in alveolar type 2 cells is consequently both a physiological device for surfactant economy and a vulnerability that converges on idiopathic pulmonary fibrosis (IPF) and acute respiratory distress syndrome.

Mitochondrial fusion is the most clearly resolved mechanistic axis. Conditional deletion of the outer-membrane GTPases MFN1 and MFN2 in murine alveolar type 2 cells causes morbidity, mortality, and spontaneous lung fibrosis, and disables the synthesis of surfactant phospholipids and cholesterol; fatty-acid-synthase deficiency in the same cells phenocopies and exacerbates bleomycin-induced fibrosis (Fan et al., 2023; Hao et al., 2024; Chung et al., 2019). This *in vivo* experiment ties surfactant lipid output directly to mitochondrial fusion machinery that, in non-pulmonary tissues, also organises the LDMC interface, and supports the interpretation that surfactant assembly is coupled to mitochondrial fusion machinery—although the lamellar body itself is a lysosome-related organelle rather than a lipid droplet, so this coupling is a distinct organelle interaction rather than an LDMC in the strict sense. Within the lamellar body itself, surfactant protein SP-B drives membrane disc formation in the trans-Golgi network and shapes the multilamellar lattice via direct lipid–protein interaction, establishing a molecular template on which the LDMC machinery operatescontact-site biology. Within the lamellar body itself, surfactant protein SP-B drives membrane disc formation in the trans-Golgi network and shapes the multilamellar lattice via direct lipid–protein interaction, establishing a molecular template on which the LDMC machinery operates (Cañadas et al., 2020; Sever et al., 2021; Sun et al., 2024). Trafficking of the flippase ATP8A1 to lamellar bodies is in turn directed by the AP-3 sorting complex, and disruption of the AP-3/ATP8A1 interaction causes ATP8A1 retention in early or recycling endosomes, increases phosphatidylserine exposure on the cytosolic leaflet, and aberrantly activates the transcriptional coactivator Yes-associated protein (YAP), augmenting alveolar type 2 cell migration and number (Kook et al., 2021). This lamellar-body trafficking defect connects a lysosome-related-organelle delivery error to a YAP-driven repair programme that is genetically linked to Hermansky–Pudlak-syndrome pulmonary fibrosis, illustrating how an upstream LDMC-adjacent failure can rewire alveolar repair (Zeng and Yan, 2025).

Lipid metabolism within alveolar type 2 cells is also tightly coupled to mitochondrial ketogenesis. Downregulation of the mitochondrial ketogenic enzyme 3-hydroxy-3-methylglutaryl-CoA synthase 2 (HMGCS2) in alveolar type 2 cells drives lipid accumulation, generates lysophosphatidylcholine species that activate adjacent fibroblasts, and accelerates pulmonary fibrosis; alveolar-type-2-specific HMGCS2 loss reproduces the fibrotic phenotype in mice (Yang et al., 2024; Chen X. et al., 2023). This places mitochondrial ketogenesis at the LDMC interface as a paracrine signalling output to fibroblasts. A complementary disease axis emerges in obesity-associated acute lung injury. High-fat-diet obesity combined with hyperoxia induces alveolar-type-2 intracellular lipid accumulation and increases CPT1A expression, yet transcriptionally downregulates mitochondrial fatty-acid oxidation and produces bioenergetic failure; alveolar-type-2-specific Cpt1a deletion exacerbates surfactant impairment and lung injury (Fan et al., 2023; Chen X. et al., 2023; Kallinos et al., 2025). The Kallinos finding is mechanistically central because it shows that elevated CPT1A protein does not equate to functional droplet-to-mitochondrion fatty-acid flow, and that the LDMC interface can be uncoupled at the regulatory level even when its core components are present. Alveolar type 2 cells therefore demand quantitative read-outs of contact-site engagement, not protein-level expression alone.

The cell-type-specific stoichiometry of PLIN5, PLIN2, MFN2, ESYT1/2, and MIGA2 in human alveolar type 2 cells, and how lamellar-body biogenesis remodels these stoichiometries during repair after viral or hyperoxic injury, has not been quantified at single-cell resolution. The PLIN5-S155 phospho-switch has not been mapped to lung-specific cues such as cyclic mechanical strain, oxygen tension, or surfactant turnover. Lipid-droplet–lamellar-body interconversion remains a structurally and mechanistically open question. The Sever lattice for lamellar-body assembly (Sever et al., 2021) and the Klein contact-site architecture (Klein et al., 2021) together provide complementary templates on which such work can be organised. The broader claim is that the alveolar type 2 cell is the most spatially complex LDMC compartment in the lung and that pharmacological engagement of the contact site here will require cell-type-precise delivery rather than systemic exposure (Pranty et al., 2026; Zeng and Yan, 2025).

#### 2.2.2 Macrophages: surfactant recycling, cholesterol overload, and infection

Macrophages are the lung’s dominant lipid-recycling compartment. Alveolar macrophages reclaim surfactant from the alveolar lumen, while interstitial macrophages survey the parenchymal lipid pool. Both compartments rely on LDMC-coupled fatty-acid handling for resolution of inflammation, and both can be redirected into a lipid-laden, foam-cell-like phenotype that supports chronic disease.

Surfactant recycling is the alveolar macrophage’s signature task. CD44 loss in mice disrupts alveolar surfactant lipid homeostasis, sensitising the lung to oxidised-lipid–induced inflammation and identifying surfactant scavenging as a quantitatively important alveolar-macrophage function (Dong et al., 2020; Lee E. et al., 2024; Morán-Lalangui et al., 2025). Alveolar macrophages are anatomically and metabolically specialised for this loop; recent integrative reviews catalogue the cell type as a guardian of an alveolar lipid galaxy whose disturbance contributes to nearly every chronic lung disease (Xie et al., 2025; Little et al., 2025; Scur et al., 2022; Hernández-Hernández et al., 2024). The aetiological landscape of lipid-laden macrophages in the lung now spans aspiration, e-cigarette injury, immune deficiency, infection, and surfactant-handling defects, and each etiology rewires the LDMC interface in characteristic ways (Stevenson et al., 2023; Guerrini et al., 2020). When the recycling loop tips toward accumulation, the resulting lipid-laden macrophage phenotype amplifies inflammatory signalling, perpetuates injury, and resists therapeutic resolution; this phenotype unifies a broad swath of chronic lung disease across chronic obstructive pulmonary disease, IPF, tuberculosis, and acute lung injury (Zhu Y. et al., 2024; Stevenson et al., 2023; Guerrini et al., 2020), with additional cohort-derived foam-cell signatures supporting this convergence (Lee E. et al., 2024).

A cholesterol-driven axis has now been formally tied to chronic obstructive pulmonary disease. Plasma metabolomics in patients with stage III–IV disease, paired with a high-cholesterol-diet plus chronic cigarette-smoke mouse model and primary macrophage assays, identifies cholesterol overload as a driver of macrophage mitochondrial dysfunction, reactive-oxygen-species production, and downstream pro-inflammatory signalling (Ran et al., 2026). The result places cholesterol metabolism upstream of mitochondrial output in chronic obstructive pulmonary disease macrophages and motivates LXR-axis and statin re-investigation as LDMC-adjacent strategies in this population (Hernández-Hernández et al., 2024; Giambelluca et al., 2025; Ding et al., 2024).

In allergic asthma, the LDMC interface is reset toward fatty-acid oxidation in a tightly regulated way. Myeloid-specific deletion of fatty-acid-binding protein 5 (FABP5) increases free long-chain unsaturated fatty acids, most notably oleic acid, in macrophages and reprograms them toward alternative activation through enhanced β-oxidation, tricarboxylic-acid-cycle flux, oxidative phosphorylation, and PPARγ signalling; the result is more severe allergen-induced airway inflammation (Hou et al., 2022; Lu et al., 2025; Wu et al., 2026). FABP5 thus operates as a checkpoint that buffers free fatty-acid loading onto the LDMC interface in macrophages, and its loss tips the balance toward an M2 programme that potentiates asthma rather than resolving it. The Hou finding is important because it shows that LDMC engagement is not unidirectionally protective; the same droplet-to-mitochondrion flow that resolves macrophage inflammation in one setting can drive pathological alternative activation in another (Lu et al., 2025; Wu et al., 2026; Huang et al., 2022).

Tuberculosis provides the most extensively dissected example of LDMC remodelling under infection. Lipid-droplet formation in *Mycobacterium* tuberculosis-infected macrophages is now understood as a host-driven, IFN-γ/HIF-1α-dependent component of the immune response rather than a bacterially imposed nutrient depot; *in vivo*, HIF-1α and its target Hig2 are required for the majority of lipid-droplet formation in the lungs of infected mice, and these immune-driven droplets restrict Mtb lipid access while supporting host-protective eicosanoid production (Knight et al., 2018; Kalam et al., 2023; Mohareer et al., 2020; Mekonnen et al., 2021). A complementary axis defines the activation-state dependence of foam-cell formation. Interleukin-4-activated macrophages resist foam-cell conversion by routing droplet-derived fatty acids into mitochondrial fatty-acid oxidation, but direct Mtb infection forces a HIF-1α-driven aerobic-glycolytic shift that produces foam-cell macrophages; murine alveolar macrophages, which exhibit predominantly fatty-acid-oxidation metabolism, are correspondingly less prone to foam-cell conversion than bone-marrow-derived macrophages (Agarwal et al., 2021; Genoula et al., 2020; Chen et al., 2024). Macrophage fatty-acid metabolism therefore arbitrates antimicrobial outcome at the cellular level, and the contact-site interface offers a regulatable lever (Kalam et al., 2023; Mohareer et al., 2020; Chen et al., 2024; Laval et al., 2021). A second-generation question is whether droplet-derived fatty-acid channelling through PLIN5–FATP4 or MFN2 is the bottleneck through which alveolar macrophages maintain their fatty-acid-oxidation-protective phenotype.

In COVID-19, the same loop is hijacked by Severe acute respiratory syndrome coronavirus 2. ORF3a redirects lipid-droplet stores into replication-organelle biogenesis through induced microlipophagy, generating novel droplet-membrane contact sites that supply viral assembly with phospholipid precursors (Li Z. et al., 2025; Dias et al., 2020; Wang et al., 2023; López-Ayllón et al., 2024) in a process dependent on host lipid-droplet remodelling (Nardacci et al., 2021). This re-purposing of LDMC machinery for viral biogenesis suggests that LDMC-engagement modulators tested in chronic lung disease could be repositioned against acute viral pneumonia, although the cell-type selectivity needed for safety remains undefined.

Interstitial macrophages constitute a distinct LDMC-relevant compartment whose biology remains under-systematised in human respiratory disease. Unlike alveolar macrophages, interstitial macrophages are predominantly monocyte-derived under inflammatory conditions, occupy the lung parenchyma, and survey perivascular and peribronchial lipid pools. Their tether and flux machinery has not been profiled in IPF, chronic obstructive pulmonary disease, or asthma cohorts, even though single-cell transcriptomic surveys identify distinct lipid-metabolic profiles between the two macrophage compartments. Resolving interstitial-macrophage LDMC biology is a precondition for cell-type-selective inhaled delivery and constitutes a parallel research line to the foam-cell discussion above.

The macrophage compartment therefore presents LDMC biology in three operational modes, namely, surfactant recycling, cholesterol-driven inflammation, and infection-driven foam-cell formation. The PFKL–PLIN2–CPT1A axis, mechanistically established in hepatocellular carcinoma but not yet resolved in lung macrophages, is an attractive candidate target in this set by analogy, because it directly couples cytosolic glycolytic flux to contact-site-resolved oxidation and admits small-molecule intervention (Giambelluca et al., 2025; Huang et al., 2022).

#### 2.2.3 Fibroblasts and myofibroblasts: A high-yield, under-resolved compartment

In the healthy lung, fibroblasts are mostly quiescent stromal cells with modest lipid reserves. After injury, however, a subset of resident interstitial fibroblasts and emergent pathological myofibroblasts undergoes a metabolic rewiring whose central feature is sustained matrix production at high energetic cost. We emphasise at the outset that no study has directly imaged or functionally quantified mitochondrion–lipid-droplet contact sites in primary lung fibroblasts; the LDMC relevance of this compartment is inferred from metabolic and transcriptomic data and from mechanisms established in other tissues. With that caveat, the bioenergetic and lipid requirements of the myofibroblast state make LDMC a plausible contributor to fibrotic biology, and the contact site has not been imaged at high resolution in primary lung fibroblasts as it has been in alveolar type 2 cells (Bezawork-Geleta et al., 2025; Klein et al., 2021).

Three converging lines of evidence motivate fibroblast LDMC as a research priority. First, single-cell transcriptomic reanalysis of idiopathic pulmonary fibrosis lungs identifies a coordinated upregulation of fatty acid oxidation, glycerolipid biosynthesis and cholesterol handling specifically in expanding myofibroblast clusters, with the strongest signal in clusters expressing collagen, smooth muscle actin and lipid-droplet scaffold genes (Bueno et al., 2020; Tian et al., 2023; Shi et al., 2024; Li W. et al., 2025). Second, mini-reviews of fatty-acid metabolism in idiopathic pulmonary fibrosis tie the same transcriptional programme to PPAR-γ/PGC-1α modulation, β-oxidation flux and aberrant prostaglandin/leukotriene signalling—pathways that depend on lipid-droplet-derived substrate delivery and mitochondrial respiratory capacity (Geng et al., 2021; Larson-Casey et al., 2020; MacIsaac et al., 2024). Third, broader synthesis of fibrotic lung disease metabolomics shows shared signatures across idiopathic pulmonary fibrosis, scleroderma-associated interstitial lung disease and post-COVID-19 fibrosis, including elevated lipid-droplet–associated transcripts and altered β-oxidation intermediates, suggesting that a common LDMC architecture supports myofibroblast persistence rather than being disease-specific (MacIsaac et al., 2024; Kumari et al., 2025; Siekacz et al., 2021; Nie et al., 2023).

A complementary signalling axis is the lysophosphatidic acid (LPA) pathway. LPA, generated from membrane lysophospholipids by the lung-enriched autotaxin (ENPP2), engages LPA receptor one on fibroblasts to drive proliferation, migration and contractile activation, and clinical-stage LPAR1 antagonism is now in late-phase idiopathic pulmonary fibrosis trials (MacIsaac et al., 2024; Nakamura and Shimizu, 2023). Although LPA signalling is conventionally framed as a G-protein-coupled cascade, the upstream LPA precursors are partitioned through lipid droplets, and the downstream Ca^2+^ and mTORC1 effectors converge on mitochondrial respiratory adaptation. The lipid-droplet–mitochondrion contact site is therefore the most parsimonious location at which LPA-driven biophysical signals are translated into the substrate availability that sustains procollagen biosynthesis and force generation. Alveolar-type-2 loss of HMGCS2, which precipitates lysophosphatidylcholine release and fibroblast activation (Yang et al., 2024), provides a paracrine link in the same direction: epithelial LDMC defects deliver lipid signals that fibroblasts must accommodate through their own contact-site biology.

The outstanding questions for fibroblast LDMC are pragmatic. Does the PLIN5-S155 phospho-switch (Benador et al., 2018; Kang et al., 2026) govern lipid mobilisation in myofibroblasts as it does in oxidative alveolar type 2 cells, and is its directionality reversed in pathological versus reparative fibroblast states? Are MIGA2-anchored endoplasmic-reticulum–lipid-droplet–mitochondria triple contacts (Freyre et al., 2019; Kim et al., 2022) enriched at sites of new collagen secretion, where the energetic cost of triple-helical assembly is greatest? Do PFKL puncta tether lipid droplets to mitochondria in lung myofibroblasts as they do in hepatocellular carcinoma cells (Meng et al., 2024), and would PFKL inhibition selectively de-energise pathological fibroblast clones without compromising regenerative repair? These questions are tractable with current imaging and conditional-genetic tools but remain unanswered, marking fibroblast LDMC as a high-yield gap and the most likely venue for repurposing nintedanib and LPAR1 antagonists with LDMC-axis modulators in idiopathic pulmonary fibrosis combination regimens, drawing on mitochondrial-quality-control and lipid-handling pathways (Larson-Casey et al., 2020; Siekacz et al., 2021; Nie et al., 2023) and IPF-specific ECM and respiratory-physiology read-outs (Li W. et al., 2025).

#### 2.2.4 The pulmonary endothelium: the field’s largest gap

The pulmonary endothelium presents the most poorly resolved compartment in the LDMC literature, and this gap is one of the central scientific observations of the present review. To state it directly: no study has demonstrated LDMC contact sites in pulmonary endothelium, and every mechanism discussed in this subsection is inferred from endothelial metabolic dependence, cross-tissue analogy, or mitochondrial-transfer therapeutics. Pulmonary microvascular endothelial cells are highly dependent on fatty-acid β-oxidation for membrane phospholipid biosynthesis during proliferation and barrier maintenance, yet whether LDMC contact sites specifically structure that dependence in the lung capillary bed remains experimentally untested with the methods available for alveolar type 2 cells, macrophages or fibroblasts (Shi et al., 2024), even though endothelial lipid-handling datasets provide a useful starting point (Lee et al., 2022; Pokharel et al., 2023).

The strongest current evidence enters indirectly, through mitochondrial-transfer therapeutics. Caldeira and colleagues demonstrated in 2025 that mesenchymal stromal cell-derived mitochondria are taken up by human pulmonary microvascular endothelial cells exposed to lipopolysaccharide or to plasma from hyper-inflammatory acute respiratory distress syndrome (ARDS) patients, restoring mitochondrial respiration and barrier integrity *in vitro* and attenuating lung injury in lipopolysaccharide-challenged mice (de Assis Fernandes Caldeira et al., 2025; Wang J. et al., 2025). The therapeutic mechanism implies that pulmonary endothelial mitochondria are limiting under inflammatory stress, and the lipid-handling capacity of recipient cells must adapt to the supplied mitochondria, but the contact-site biology has not been examined directly. Whether donor mitochondria establish productive LDMC contacts in recipient endothelium, whether endogenous PLIN-decorated lipid droplets in pulmonary endothelium ferry fatty acids to those mitochondria, and whether PFKL–PLIN2 tethering, a mechanism demonstrated only in hepatocellular carcinoma, operates in endothelium at all, and how the inflammatory milieu rewires endothelial PFKL–PLIN2 tethering are all open (Meng et al., 2024).

The case for a focused experimental campaign is strong. The pulmonary capillary bed is the obligate first-pass site for postprandial chylomicron remnants, and endothelial LDMC is a plausible regulator of pulmonary hypertension, ARDS, fibrotic vascular remodelling and tumour vasculogenesis. The minimum first set of experiments would be live-cell imaging of lipid-droplet–mitochondrion contact dynamics in primary human pulmonary microvascular endothelial cells under inflammatory and hypoxic conditions, paired with PLIN5-S155 and MIGA2 perturbations and respiratory phenotyping drawing on existing endothelial datasets (Zhang and Michelakis, 2021; Nukala et al., 2021; Flerlage et al., 2023) and pulmonary microvascular and ARDS models (Zhu L. et al., 2024; Yao et al., 2026; Wang F. et al., 2025). Without that dataset, the endothelial compartment will continue to be reasoned about by analogy rather than evidence—a state the field can no longer accept given the centrality of pulmonary vasculature to most of the diseases enumerated below (Nukala et al., 2021; Wang F. et al., 2025).

#### 2.2.5 Lung adenocarcinoma: the only lung compartment with direct LDMC evidence

Lung adenocarcinoma (LUAD) is the most common lung cancer histology and the only lung disease in which LDMC has been directly demonstrated by contact-resolving methods. Han and colleagues used spatial proteomics and electron tomography across human LUAD samples to map mitochondrial network topology and bioenergetics *in situ*, and their landmark dataset established that high-grade LUAD regions exhibit dense lipid-droplet–mitochondrion contact sites, oxidative-phosphorylation-leaning tumour cells, and a metabolic geography in which peridroplet mitochondria support cellular respiration in lipid-rich microenvironments (Han et al., 2023; Jin and Yuan, 2020; Liu et al., 2023). Separately, Meng and colleagues reported in 2024 that PFKL, long regarded as a purely glycolytic enzyme, is a bifunctional protein whose puncta tether lipid droplets to mitochondria through PLIN2 and recruit CPT1A to the contact, thereby coupling cytosolic glycolytic flux to β-oxidation and tumour growth. Critically, that mechanism was established in hepatocellular carcinoma, not in lung cancer; its relevance to LUAD is inferred from the shared peridroplet mitochondrial geography mapped by Han and colleagues, and it has not been experimentally demonstrated in lung tissue. The PFKL–PLIN2–CPT1A axis is arguably the most experimentally complete LDMC tether-and-flux mechanism in any disease context, but it was established in liver, not lung: PFKL puncta physically dock PLIN2-decorated lipid droplets to mitochondria, recruit CPT1A and ATGL, and channel free fatty acids into β-oxidation (Meng et al., 2024). If this dual role operates in LUAD, it could help explain why glycolysis-targeted therapy has had limited efficacy in oxidative-phosphorylation-leaning tumours: the same enzyme also organises the contact-site machinery that supports oxidative metabolism. Testing that inference in lung models is a priority experiment.

Several streams of evidence implicate the perilipin family directly in LUAD progression. Miyata-Morita and colleagues demonstrated in human LUAD cohorts that high PLIN2 expression associates with poor differentiation, blood-vessel and pleural invasion, advanced stage and shorter recurrence-free survival, and that CRISPR/Cas9 PLIN2 knockout in A549 and PC-9 cells reduces lipid-droplet accumulation, suppresses oleic-acid–induced lipid-droplet formation, and impairs proliferation and migration (Gründing et al., 2022; Miyata-Morita et al., 2025). PLIN3, in a pan-cancer analysis with focused LUAD validation, is upregulated in tumours, correlates with adverse prognosis, and recruits M2 tumour-associated macrophages, establishing a paracrine axis through which tumour LDMC restructures the immune microenvironment (Torresano et al., 2022; Yang et al., 2025). Earlier *in vitro* work showed that lipid droplets themselves are critical for LUAD cell growth and starvation survival, indicating that perilipin-decorated lipid-droplet stores function as substrate reservoirs rather than as inert lipid sinks (Lung et al., 2022).

CPT1A is the prevailing β-oxidation entry point in LUAD LDMC. Ma and colleagues, using a lung-epithelium-specific Cpt1a knockout mouse, demonstrated that CPT1A suppresses ferroptosis in cancer stem cells through a c-Myc-positive feedback loop that activates the NRF2/GPX4 axis and downregulates ACSL4, and that pharmacological CPT1A inhibition synergises with immune checkpoint blockade to enhance both anti-tumour immunity and tumoural ferroptosis (Ma et al., 2024). Xu and colleagues complemented this finding by showing that miR-365-3p, downregulated in lung cancer, normally suppresses CPT1A translation; loss of miR-365-3p drives the lipid-droplet–CPT1A–fatty-acid-oxidation axis to support proliferation and migration, and rescue experiments confirm that the proliferative phenotype is fatty-acid-oxidation-dependent (Xu et al., 2025; De Oliveira and Liesa, 2020). Together, these genetic and pharmacological data place CPT1A at the centre of a candidate LDMC vulnerability in LUAD, demonstrated at the level of CPT1A-dependent fatty-acid oxidation and ferroptosis resistance, with the contact-site tether itself inferred from hepatocellular carcinoma, that interlocks with two of the most active areas in lung cancer therapeutics: immune checkpoint inhibition and ferroptosis induction.

A translational precedent exists. Cai and colleagues constructed and validated a lipid-droplet–mitochondria-associated risk score from eight genes that stratifies LUAD survival and immunotherapy response in TCGA cohorts, providing a precedent for LDMC-derived clinical decision tools (Cai et al., 2024). Reviews of broader lipid-metabolism reprogramming in epidermal growth factor receptor (EGFR)-mutated non–small cell lung cancer also implicate fatty acid synthase, stearoyl-CoA desaturase and sterol regulatory element-binding protein in tyrosine kinase inhibitor resistance, and these pathways converge on lipid-droplet biogenesis and remodelling (Eltayeb et al., 2022). The unresolved question is whether the PFKL–PLIN2–CPT1A axis acts as the master regulator of these downstream effectors or operates as one of several parallel routes, a distinction whose resolution determines whether single-target or combination LDMC therapy is the appropriate translational strategy in LUAD. Together, the LUAD evidence base shows that LDMC is directly demonstrated in lung cancer by spatial proteomics and electron tomography, while the central tether-and-flux mechanism (PFKL–PLIN2–CPT1A) remains extrapolated from hepatocellular carcinoma; LUAD therefore provides the most mature, but still partly inferential, template against which other lung diseases are benchmarked.

### 2.3 The disease matrix: cell-type-resolved failure modes across respiratory illness

**FIGURE 3:**
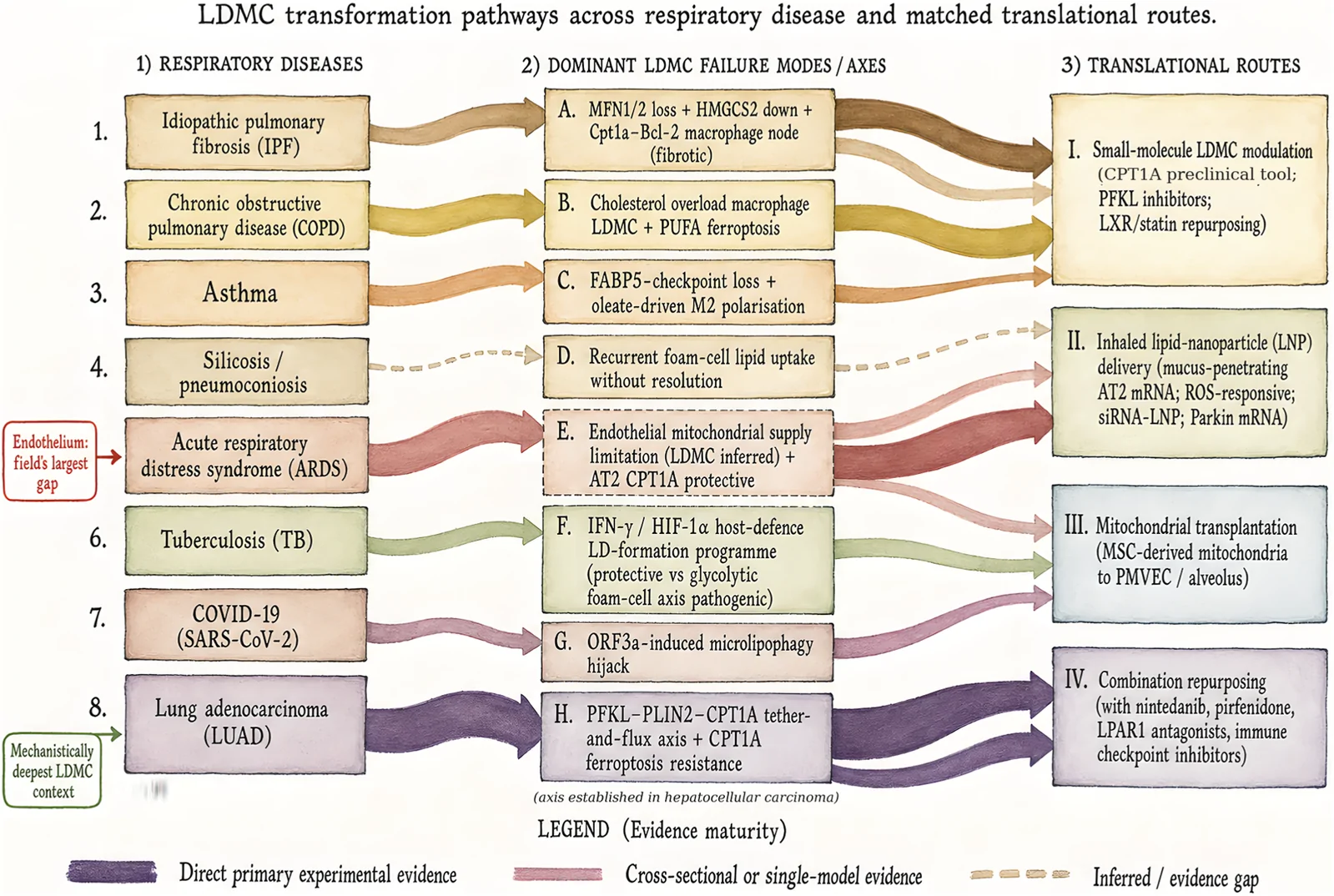
Disease-to-failure-mode-to-therapy Sankey map of the pulmonary LDMC landscape. Left column, eight respiratory diseases. Middle column, eight LDMC failure modes (A–H). Right column, four operational therapeutic routes. Ribbon width is illustrative of the breadth of published mechanistic evidence, not a quantitative flow; ribbon shading distinguishes failure modes supported by direct LDMC evidence (contact-resolving measurement in lung; currently only mode H, via LUAD spatial proteomics) from those supported by indirect lung evidence or cross-tissue analogy. Failure modes: A, MFN1/MFN2 loss and HMGCS2 downregulation in AT2 cells, with the Cpt1a–Bcl-2 apoptosis-resistance axis in lung macrophages (IPF); B, cholesterol overload and PUFA-triggered ferroptosis in alveolar macrophages (COPD); C, FABP5-checkpoint loss with oleate-driven M2 polarisation (asthma); D, recurrent exogenous lipid uptake with foam-cell accumulation (silicosis); E, endothelial mitochondrial supply limitation with alveolar-barrier collapse (ARDS; LDMC involvement inferred); F, IFN-γ/HIF-1α–driven host-defence droplet biogenesis and granuloma-associated lipid handling (tuberculosis); G, ORF3a-driven microlipophagy hijack for viral replication-organelle biogenesis (COVID-19); H, PFKL–PLIN2–CPT1A tether-and-flux axis with CPT1A-mediated ferroptosis resistance (LUAD; the PFKL tether mechanism is established in hepatocellular carcinoma and inferred in lung). Therapeutic routes: (i) small-molecule modulation of contact-site tethers and flux nodes (CPT1A tool inhibitors, PFKL, LXR, statins); (ii) inhaled mucus-penetrating LNP delivery of ROS-responsive/siRNA/Parkin mRNA payloads; (iii) mesenchymal-stem-cell mitochondrial transplantation; (iv) combination repurposing of nintedanib, pirfenidone, LPAR1 antagonists and immune-checkpoint inhibitors with LDMC-axis modulators.

The same LDMC machinery acquires disease-specific configurations across the spectrum of respiratory illness. This synthesis identifies, for each disease, the dominant LDMC failure mode, the most experimentally tractable cell-type entry point, and the therapeutic question that emerges. The full disease-to-failure-mode-to-therapy matrix is presented as a Sankey diagram in Figure 3 and expanded row-by-row in Table 3, with representative references retained per disease.

Idiopathic pulmonary fibrosis (IPF). IPF combines alveolar-type-2 mitochondrial dysfunction, alveolar-type-2-derived lipid signalling to fibroblasts, and a macrophage compartment that resists apoptosis. Mitofusin-mediated alveolar-type-2 lipid metabolism failure drives spontaneous fibrosis in mice (Chung et al., 2019), HMGCS2 downregulation in alveolar type 2 cells releases lysophosphatidylcholine and activates fibroblasts (Yang et al., 2024), and alveolar-type-2 mitochondrial permeability transition pore opening propagates the injury (Larson-Casey et al., 2020; MacIsaac et al., 2024; Lu et al., 2024). In the macrophage compartment, Cpt1a interacts with mitochondrial Bcl-2 in IPF lung macrophages to drive apoptosis resistance and fibrotic remodelling, providing the most explicit macrophage-LDMC mechanism in chronic lung disease (Gu et al., 2022). The dominant failure mode is therefore distributed across alveolar, mesenchymal and macrophage compartments rather than localised to a single cell type, which explains why monotherapy with nintedanib or pirfenidone slows but does not arrest disease (Bonella et al., 2023). The most tractable LDMC intervention is the Cpt1a–Bcl-2 axis in macrophages paired with alveolar-type-2 mitochondrial protection (Wang et al., 2024a). Mechanistically, Cpt1a binds the BH3 domain of mitochondrial Bcl-2 to suppress macrophage apoptosis, sustaining the lipid-laden macrophage population that drives fibrogenic crosstalk with alveolar type 2 cells and fibroblasts (Gu et al., 2022) (Siekacz et al., 2021). The implication is that anti-apoptotic CPT1A–Bcl-2 disruption could deplete pro-fibrotic macrophages without targeting epithelial CPT1A (MacIsaac et al., 2024).

Chronic obstructive pulmonary disease (COPD). COPD pathogenesis converges on cigarette-smoke-driven oxidative stress, alveolar macrophage dysfunction, and cholesterol-driven metabolic reprogramming. Multi-omics analysis of human COPD samples integrated with cigarette-smoke and high-cholesterol-diet mouse models established cholesterol-driven macrophage mitochondrial dysfunction, reactive oxygen species generation, and downstream inflammation as a quantitatively dominant axis (Ran et al., 2026). Recent synthesis positions ferroptosis, driven by iron-catalysed peroxidation of polyunsaturated fatty acids in membrane phospholipids and amplified by DRP1-mediated mitochondrial fission and lipid-droplet–mitochondria contact remodelling, as a unifying mediator of COPD pathology (Ni et al., 2026; Manevski et al., 2020). The COPD foam-cell phenotype is part of a unifying lipid-laden-macrophage state that also encompasses tuberculosis, silicosis and acute lung injury (Manevski et al., 2020). The cell-type entry point is the alveolar macrophage; the therapeutic question is whether LDMC stabilisation upstream of ferroptosis can be achieved without compromising clearance of inhaled lipid loads (Fairley et al., 2023).

Asthma. Asthma extends the macrophage LDMC narrative into adaptive-immune-driven disease. FABP5 deletion in myeloid cells enhances IL-4-driven M2 polarisation and aggravates allergic airway inflammation in murine models, with the mechanism traced to oleic-acid accumulation, fatty-acid-oxidation upregulation, tricarboxylic-acid-cycle activation and PPAR-γ signalling (Wang et al., 2026). Asthmatic macrophages therefore present an LDMC state that is permissive for, rather than resistant to, allergic inflammation, and the dominant failure mode is dysregulated lipid trafficking rather than impaired bioenergetics. The therapeutic question is whether selective restraint of macrophage fatty-acid oxidation can attenuate exacerbations without disrupting host defence, which itself depends on macrophage lipid handling (Esteves et al., 2021; Ravi et al., 2021), and viral and asthma-cohort macrophage profiles (Hartsoe et al., 2024; Ni et al., 2025).

Silicosis and pneumoconiosis. Inhalation of mineral dusts produces persistent alveolar macrophage activation and lipid-laden macrophage accumulation that resemble the COPD and tuberculosis foam-cell phenotypes but are sustained by chronic exogenous lipid input. The dominant failure mode is recurrent lipid uptake without effective resolution: the alveolar macrophage compartment cannot complete the surfactant-recycling loop. The therapeutic question is whether LDMC stabilisation could shift mineral-laden macrophages toward a pro-resolution state and prevent progression to fibrotic remodelling (Adamcakova and Mokra, 2021).

Acute respiratory distress syndrome (ARDS). ARDS is dominated by acute endothelial barrier failure superimposed on alveolar epithelial injury, with mitochondrial dysfunction and pulmonary oedema as principal mediators (Robinson and Krasnodembskaya, 2020). The strongest LDMC-adjacent therapeutic evidence is in this disease. Pulmonary endothelial mitochondrial dysfunction induced by lipopolysaccharide or by hyper-inflammatory ARDS patient plasma is reversed by transplantation of mesenchymal-stromal-cell-derived mitochondria, restoring barrier integrity in primary human pulmonary microvascular endothelial cells and attenuating injury in lipopolysaccharide-challenged mice (de Assis Fernandes Caldeira et al., 2025). A complementary line of work demonstrated that mitochondrial transplantation alleviates lipopolysaccharide-induced ARDS through reduction of alveolar inflammation and apoptosis (Lee S-E. et al., 2024). CPT1A overexpression in alveolar epithelium also alleviates lipopolysaccharide-induced acute lung injury, identifying CPT1A as a candidate pharmacological target (Wang G-Y. et al., 2024; McClintock et al., 2022; Shen et al., 2024). The cell-type entry point is the pulmonary microvascular endothelium and the alveolar type 2 cell in parallel (Flerlage et al., 2023; Wang F. et al., 2025).

Tuberculosis. *Mycobacterium tuberculosis* exploits and rewires macrophage LDMC. Lipid-droplet formation in infected macrophages is not pathogen-driven but is an interferon-γ-induced, HIF-1α–dependent host-defence programme required for granuloma function (Kalam et al., 2023), while direct mycobacterial infection counteracts the alternative-activation fatty-acid-oxidation programme that would otherwise prevent foam-cell formation, driving glycolytic metabolic reprogramming and tissue-damaging lipid accumulation (Shim et al., 2020). Macrophage fatty-acid metabolism therefore arbitrates infection outcome. The therapeutic question is whether host-directed therapy that nudges LDMC toward the IFN-γ/HIF-1α-protective configuration could shorten antibiotic-treatment duration without rebound inflammation. Alveolar macrophages appear less prone to glycolytic foam-cell conversion than bone-marrow-derived macrophages, raising the possibility of compartment-specific effects: an LDMC modulator may benefit interstitial macrophage states without disturbing alveolar macrophage host defence (Genoula et al., 2020; Laval et al., 2021). This cell-type stratification has not been incorporated into any tuberculosis host-directed therapy trial (Chen et al., 2024; Shim et al., 2020).

COVID-19. SARS-CoV-2 hijacks lipid-droplet–mitochondria flux through ORF3a-induced microlipophagy to deliver lipid substrates for replication-organelle biogenesis, demonstrating a viral subversion of LDMC architecture. Broader profiling of metabolic alterations after SARS-CoV-2 infection has mapped extensive remodelling of lipid metabolism, fatty-acid biosynthesis, and mitochondrial function, identifying multiple potential therapeutic targets (Chen P. et al., 2023; Cevallos et al., 2025; Park, 2025). The dominant failure mode is acute viral co-option, and the therapeutic question is whether host LDMC stabilisation could reduce viral fitness without compromising the antiviral interferon response (D’Avila et al., 2024).

Lung adenocarcinoma (LUAD). LUAD is the only disease in the matrix with direct LDMC evidence (spatial proteomics and electron tomography of peridroplet mitochondria), and it serves as the benchmark for what cell-type-resolved LDMC therapy looks like when the molecular pieces are in place while noting that its central tether mechanism (PFKL–PLIN2–CPT1A) is extrapolated from hepatocellular carcinoma: PLIN2 as a prognostic biomarker (Miyata-Morita et al., 2025), PLIN3 as a tumour-associated-macrophage-interfacing target, CPT1A as a ferroptosis-resistance node (Ma et al., 2024), the PFKL–PLIN2–CPT1A flux axis (established in hepatocellular carcinoma, inferred in LUAD) as a candidate central regulator, and an eight-gene lipid-droplet–mitochondria risk score that already stratifies patients in TCGA cohorts (Cai et al., 2024). Outside LUAD, the matrix is dominated by gaps that the next section translates into a programmatic translational pipeline.

## 3 Translational pipeline

The cell-type-resolved LDMC mechanisms summarised above, graded by evidence tier, support an integrated translational research programme rather than a list of disconnected hypotheses. Four therapeutic routes have preclinical proof of concept in respiratory disease: small-molecule modulation of contact-site tethers and flux nodes, inhaled lipid-nanoparticle delivery of LDMC-targeted payloads, mitochondrial transplantation, and combination repurposing of approved antifibrotics or immune checkpoint inhibitors with LDMC-axis modulators. Each route carries a specific safety constraint that should shape further development (Wan et al., 2023; Al-Jipouri et al., 2023; Zong et al., 2024).

### 3.1 Four operational routes

Small-molecule LDMC modulation. The PFKL–PLIN2–CPT1A axis is the most experimentally complete LDMC pharmacological target in principle, but its evidence base is split: the molecular tether is mapped in hepatocellular carcinoma (not lung), whereas the catalytic CPT1A node is demonstrated in LUAD models and addressable with preclinical tool inhibitors such as etomoxir (Wang G-Y. et al., 2024), and the downstream lipid-droplet–mitochondria risk score validated in LUAD cohorts (Ma et al., 2024; Cai et al., 2024). CPT1A inhibition in tumour models synergises with immune checkpoint blockade through ferroptosis induction (Ma et al., 2024), and CPT1A overexpression alleviates lipopolysaccharide-induced acute lung injury (Wang G-Y. et al., 2024), framing CPT1A modulation as a context-dependent therapy whose direction of perturbation must match disease context (Zong et al., 2024). The Cpt1a–Bcl-2 interaction in lung macrophages provides an additional macrophage-selective target with anti-fibrotic potential (Gu et al., 2022). The corresponding safety constraint is bidirectional. Off-target CPT1A inhibition could compromise epithelial barrier defence, where higher CPT1A is protective in acute lung injury (Wang G-Y. et al., 2024), while necessary for anti-tumour or anti-fibrotic effects, so cell-type-selective delivery and on-tissue pharmacodynamic biomarkers are required. A further constraint is pharmacological: etomoxir itself is not clinically accessible. At the concentrations commonly used to inhibit CPT1A it induces severe oxidative stress and CPT1A-independent off-target effects, including direct inhibition of mitochondrial respiration, and it has never advanced beyond experimental use (Al-Jipouri et al., 2023; Zong et al., 2024; K and Narayansamy, 2025; O’Connor et al., 2018).

Inhaled lipid-nanoparticle delivery. The inhaled route is uniquely suited to LDMC pharmacology in the lung because it concentrates drug at the relevant cell types while limiting systemic exposure. Two preclinical proof-of-concept studies anchor the route. Mucus-penetrating inhalable lipid nanoparticles co-delivering messenger RNAs encoding cytochrome b5 reductase three and bone morphogenetic protein four to alveolar type 2 cells restore stemness and trigger realveolarisation in bleomycin-induced mouse fibrosis, the most direct demonstration that LDMC-adjacent epithelial biology can be reprogrammed *in vivo* (Wan et al., 2023; Bai et al., 2024). A separate nanomedicine targeting the mitochondrial permeability transition pore through coordinated cyclophilin D inhibition (by a cyclosporin-A-derived ionisable lipid) and mitochondrial calcium uniporter silencing achieves “double braking” on alveolar-type-2 mitochondrial dysfunction in IPF mouse models (Liu et al., 2022; Li D. et al., 2023; Wang et al., 2024c). Inhaled specialised pro-resolving mediator-based nanotherapeutics target the macrophage lipid-droplet–NLRP3 axis and the epithelial reactive-oxygen-species–TGF-β/Smad pathway in parallel, attenuating bleomycin- and engineered-nanomaterial-induced fibrosis (Liu et al., 2022; Li D. et al., 2023; Li J. et al., 2023; Bai et al., 2022), with additional inhaled-fibrosis nanotherapeutic platforms in active development (K and Narayansamy, 2025; Deng et al., 2025). The safety constraint is alveolar deposition kinetics in fibrotic lungs, where mucus and matrix barriers may divert payload from intended cells; head-to-head pharmacokinetic studies in human *ex vivo* lung tissue are the immediate gap (Wan et al., 2023; Al-Jipouri et al., 2023; K and Narayansamy, 2025; Zimmermann et al., 2022).

Mitochondrial transplantation. Two independent preclinical lines support the transplantation route in ARDS. Mesenchymal-stromal-cell-derived mitochondria are taken up by human pulmonary microvascular endothelial cells and restore both mitochondrial respiration and barrier integrity *in vitro* and in lipopolysaccharide-challenged mice (de Assis Fernandes Caldeira et al., 2025); exogenous mitochondrial transfer alleviates lipopolysaccharide-induced ARDS through suppression of alveolar inflammation and epithelial/endothelial apoptosis (Lee S-E. et al., 2024). The mechanism is mitochondrial replacement, but the lipid-handling capacity of recipient cells must accommodate the supplied mitochondria, raising LDMC questions that have not yet been addressed in transplantation studies. The safety constraint is donor mitochondrial mismatch (mitochondrial DNA heteroplasmy effects), pulmonary embolism risk from particulate delivery, and the unknown durability of effect. Phase one studies in adult ARDS have been proposed on the basis of manufacturing and regulatory groundwork laid for paediatric cardiac surgery transplantation (Wang et al., 2024c).

Combination repurposing. The deepest translational lever is the combination of approved or late-stage agents with LDMC-axis modulators. Nintedanib and pirfenidone slow but do not arrest IPF progression (Bonella et al., 2023), and the LDMC literature now identifies alveolar-type-2 mitochondrial protection and Cpt1a–Bcl-2 disruption in macrophages as biologically complementary mechanisms, supported by mRNA- and antifibrotic-LNP combination strategies (Bai et al., 2022; Deng et al., 2025) and ROS-responsive or pro-resolution platforms (Li D. et al., 2023; Wang et al., 2024c). LPA-receptor antagonism, an active area of late-stage IPF drug development, is positioned within the LPA–fibroblast axis that is itself coupled to lipid-droplet–mitochondria–endoplasmic-reticulum tripartite contacts and to alveolar-type-2 epithelial dysfunction. Immune checkpoint blockade in LUAD remains response-limited, and CPT1A inhibition synergises through ferroptosis induction, providing the prototype for LDMC-targeted combination immuno-oncology. Tension-induced organelle stress is emerging as a tractable mechanism in fibrosis with overlap to LDMC at the contact-site mechanical interface (Kai et al., 2025), and ARDS metabolic targeting is moving from concept toward early clinical translation (Robinson and Krasnodembskaya, 2020; Wang G-Y. et al., 2024). The safety constraint across all combinations is overlapping toxicity at the mitochondrion, where simultaneous perturbation of multiple respiratory or LDMC nodes can precipitate respiratory failure rather than fibrosis arrest. Sequential rather than concurrent dosing, with mitochondrial respiratory-reserve monitoring as a pharmacodynamic biomarker, is the conservative path forward.

## 4 Discussion

The synthesis assembled across the preceding sections converges on a single view. The lipid droplet and mitochondrion, once studied as two separate compartments of energy economy, are now best understood as a single addressable interface whose molecular composition, phosphorylation logic and spatial geometry can be enumerated, perturbed and quantified in living cells (From the American Association of Neurological Surgeons AANS et al., 2018). That interface belongs to a broader class of inter-organelle contact sites whose recognition as pharmacologically tractable interfaces has moved beyond exploratory imaging into a systematic research programme, and consensus criteria now exist for what counts as a defined contact, how it should be measured and how it should be reported (Calì et al., 2025). Within this framework the alveolus offers a rare configuration where every relevant cell type engages the same interface with a distinct stoichiometry and outcome, so that a single molecular axis can appear cytoprotective, homeostatic, immunopermissive or oncogenic depending on the compartment in which it operates. The pharmacological consequences are already being tested through a modest but growing catalogue of small molecules that either restore, tighten or dissolve specific contacts, and the design principles that underlie their selectivity are becoming articulable rather than empirical (Ko et al., 2026). What remains missing is not a further list of molecular players, since the field is now data-rich, but an integrating framework that couples flux measurement to spatial context so that the interface can be evaluated in whole tissues under disease-relevant perturbation (Mathiowetz and Olzmann, 2024). The chapters below take up that missing framework by asking what the interface still hides at the mechanistic level, where the current experimental paradigm is falling short, and how those two constraints will shape the next decade of translation.

The mechanistic ground under this interface, though far better characterised than 5 years ago, still hides several questions whose resolution will constrain any therapeutic development that follows. Foremost among them is the compartmental duality of CPT1A, the enzyme that authorises fatty acid entry into the mitochondrial matrix. In alveolar macrophages under acute injury the IL-10-linked CPT1A programme restrains inflammatory activation and limits epithelial damage (Wang M. et al., 2024), whereas in tumour and chronically-stressed macrophage settings the same enzyme sustains membrane integrity, blocks ferroptosis and supports survival despite ongoing lipid overload (Shi et al., 2025). Whether the two phenotypes reflect a shared catalytic activity read out through divergent downstream signalling, or a genuinely different flux regime driven by cell-type-specific co-factors and isoform partitioning, cannot be resolved without single-cell lipid-flux measurement that treats compartment and time as first-class variables. Related uncertainty attaches to phosphorylation-based tether regulation, where site-level information such as PLIN5 S155 occupancy governs whether a droplet feeds or sequesters lipid, yet genotype and steady-state transcript readouts collapse this dimension entirely; only through recent gains in phosphoproteomic sensitivity can stoichiometry now be tracked across subcellular fractions with the confidence needed for causal interpretation (Muneer et al., 2025; Lancaster et al., 2024). Answering these questions in native human tissue further depends on whether the transcriptional signatures already reported in lung adenocarcinoma correspond to genuine, perturbation-sensitive drug targets rather than incidental high-expression correlates. Patient-derived organoids from resected material and pleural fluid now generate perturbation readouts in clinically actionable timescales and preserve enough of the native metabolic wiring to test tether-level hypotheses in matched genetic backgrounds (Lee S-H. et al., 2024). Mechanistic ambition therefore no longer outstrips experimental capacity, but the remaining bottleneck sits at the paradigmatic level rather than at the level of individual assays.

Three features of the current experimental paradigm limit how quickly mechanistic insight will convert into clinical use, and each admits a specific methodological remedy that the field is now beginning to adopt. Species mismatch is perhaps the most consequential of these, because mouse and human lungs diverge in alveolar architecture, surfactant lipid composition and epithelial repair trajectories, and spatially-resolved comparisons of transcriptomic and cell-state maps have made those divergences quantifiable rather than anecdotal (Franzén et al., 2024). What follows is that a pharmacologically tractable node identified in a mouse model of fibrosis or macrophage lipotoxicity should be re-tested in a human air-liquid interface or organoid preparation before any target-validation claim is made, and the case for parallel-species pipelines rather than sequential-species pipelines is now backed by explicit road-map proposals (Cantor, 2025). Dimensional undersampling introduces a distinct but equally serious distortion, since membrane contact sites are three-dimensional geometries with a defined intermembrane gap and an area that cannot be recovered from single confocal slices; expansion microscopy and volumetric electron approaches now provide the resolution needed to score contact area and stoichiometry directly, and consensus workflows increasingly recommend that any imaging-based tether claim be corroborated across two orthogonal modalities before biological weight is assigned (Diokmetzidou and Scorrano, 2025; Zhuang et al., 2026; Marshall et al., 2023). Compounding both problems, cell-type-specific tether configurations are averaged out in bulk transcriptomics, so signatures derived from whole-lung homogenates conflate donor cells that behave in opposite metabolic directions, a limitation surfaced most clearly for alveolar macrophage lipid programmes where subset-level heterogeneity carries most of the disease signal (Apte et al., 2026). Aligning assay resolution with biological resolution therefore becomes as decisive as any additional mechanistic discovery for what follows, which is the translation of these interfaces into patient benefit.

Patient benefit will depend on several interlocking developments, and their order is set by scientific readiness rather than by ambition. Delivery is the most immediately tractable of these. Inhaled lipid nanoparticles offer a route to alveolar cells that bypasses first-pass metabolism, yet the endogenous surfactant film redistributes cargo and imposes screening demands that most efficacy studies still under-report; formulations that co-engineer particle surface and surfactant interaction, and that measure delivery quantitatively before invoking efficacy, are now moving from proof of concept into pulmonary-relevant preclinical work (Kassab et al., 2023; Falahati et al., 2023; Gonsalves and Menon, 2024). Trial architecture must move in step with delivery, because responder heterogeneity in interstitial and airway diseases has been the principal reason that mechanistically-plausible agents have failed at Phase II or III when tested against unstratified cohorts. Adaptive platforms that permit multiple arms, biomarker-enrichment stages and Bayesian response-adaptive randomisation are now operational for fibrotic lung disease and provide the trial-design substrate into which lipid-mitochondria interface signatures can be embedded (Kawano-Dourado et al., 2024). Beneath both delivery and trial design, precision-medicine work in idiopathic pulmonary fibrosis has moved from taxonomic descriptions of endotype toward composite molecular scores intended for enrichment, so that any interface-derived signature must fit into this framework if it is to become a companion diagnostic rather than a research instrument (Karampitsakos et al., 2023). Underpinning all of the above, biomarker maturity itself will depend on pre-analytic discipline in bronchoalveolar lavage, because the dilution problem, storage stability and cell-type composition have historically defeated attempts to compare lavage-derived measurements across sites; consensus normalisation and standardisation frameworks are now proposed and warrant broad adoption before any Phase Ib trial invokes lavage-based stratification (Haeger et al., 2024; Mawer et al., 2026). Aligning delivery, design, framework and pre-analytic rigour is what will convert the interface catalogued here into a clinical instrument, and each of these components is now defensible on published evidence rather than aspirational.

Biomarker stratification for LDMC-directed trials divides into validated and exploratory candidates, distinguished by sample type and assay. Two biomarkers are validated in the strict sense of reproducible association with clinical outcome in patient cohorts. PLIN2, measurable by immunohistochemistry or transcriptomics in resected tumour or biopsy tissue, stratifies differentiation, invasion and recurrence-free survival in lung adenocarcinoma. The eight-gene lipid-droplet-mitochondria risk score, computed from bulk tumour transcriptomes, stratifies survival and immunotherapy response in TCGA lung adenocarcinoma cohorts. All remaining candidates are exploratory. PLIN5-S155 phosphorylation occupancy would require a phospho-specific antibody assay applied to bronchoalveolar lavage cells or transbronchial biopsy, and has not been validated in any lung cohort. Contact-site occupancy itself is measurable only by imaging, whether super-resolution or electron microscopy of biopsy material, and is not yet standardised across sites. Mitochondrial respiratory reserve, measurable by extracellular-flux assay in live cells from lavage or biopsy, is a functional pharmacodynamic readout rather than a contact-site-specific one. We recommend that early-phase LDMC trials embed the two validated biomarkers for patient selection and carry the exploratory set as secondary pharmacodynamic endpoints, so that biomarker maturity can advance in parallel with the therapeutics.

Several limitations qualify the synthesis presented here. Direct evidence in human respiratory tissue remains scarce, since only one dataset, spatial proteomics with electron tomography in non-small cell lung cancer (Han et al., 2023; Jin and Yuan, 2020; Liu et al., 2023), resolves lipid-droplet–mitochondria contacts in lung; cryogenic correlative microscopy in alveolar type 2 cells (Klein et al., 2021) resolves lamellar-body–mitochondria juxtaposition, a distinct organelle interaction that we do not classify as LDMC, and most mechanisms discussed are extrapolated from metabolic or transcriptomic data or from other tissues. Quantifying functional contacts is also technically difficult, because membrane contact sites are three-dimensional and dynamic geometries, and no standardised assay yet reports contact-site number, area or flux in primary human lung cells. Even where imaging is available, colocalization by light microscopy is not evidence of a functional contact, and several lung studies cited here rely on proximity signals that have not been corroborated by orthogonal ultrastructural or flux-based methods. The pharmacological tools introduce a further caveat, in that etomoxir, the most widely used CPT1A inhibitor, exhibits dose-dependent off-target effects including oxidative stress and direct respiratory inhibition at commonly used concentrations, so conclusions drawn from it require independent genetic confirmation (Yao et al., 2018). Finally, cell-state and disease-stage variability across macrophage subset, fibroblast activation state and tumour grade means that the LDMC configurations catalogued here are likely to shift within a given disease, and most cited studies capture only a single state. These limitations define the agenda of Section 4 as much as the discoveries do.

## 5 Conclusion

LDMC has moved from a descriptive cell-biological curiosity into a mechanistically tractable, cell-type-resolved interface with emerging pharmacological relevance. Its application to respiratory medicine, however, remains uneven, and three specific gaps determine what comes next. The pulmonary endothelium is the field’s largest evidence gap, since no primary study has resolved contact-site dynamics in human pulmonary microvascular endothelial cells under inflammatory, hypoxic or fibrotic stress, and closing that gap is now within experimental reach. Beyond it, the lipid-mechanics interface remains unformalised: contact-site geometry stands at the point at which cytoskeletal tension could be transduced into lipid-flux change, but this transduction has not yet been formally tested in lung fibroblasts. Beyond both, operational biomarkers are still missing, because the field lacks validated readouts of phospho-switch occupancy or interface engagement that survive translation into bronchoalveolar lavage, sputum or biopsy material. A coordinated programme that closes these gaps would convert LDMC from a productive area of basic discovery into the cell-type-resolved therapeutic discipline that lung-disease patients have not yet benefited from in the way that liver-disease patients are beginning to. The molecular toolkit is in place, the disease anchors are mapped, the translational routes are identified, and the remaining work is execution.

## References

[B1] Adamcakova J. Mokra D. (2021). New insights into pathomechanisms and treatment possibilities for lung silicosis. Int. J. Mol. Sci. 22, 4162. 10.3390/ijms22084162 33920534 PMC8072896

[B2] Agarwal P. Gordon S. Martinez F. O. (2021). Foam cell macrophages in tuberculosis. Front. Immunol. 12, 775326. 10.3389/fimmu.2021.775326 34975863 PMC8714672

[B3] Agudelo C. W. Samaha G. Garcia-Arcos I. (2020). Alveolar lipids in pulmonary disease. A review. Lipids Health Dis. 19, 122. 10.1186/s12944-020-01278-8 32493486 PMC7268969

[B4] Al-Jipouri A. Almurisi S. H. Al-Japairai K. Bakar L. M. Doolaanea A. A. (2023). Liposomes or extracellular vesicles: a comprehensive comparison of both lipid bilayer vesicles for pulmonary drug delivery. Polym. (Basel) 15, 318. 10.3390/polym15020318 36679199 PMC9866119

[B5] Aloe C. A. Leong T. L.-T. Wimaleswaran H. Papagianis P. C. McQualter J. L. McDonald C. F. (2022). Excess iron promotes emergence of foamy macrophages that overexpress ferritin in the lungs of silicosis patients. Respirology 27, 427–436. 10.1111/resp.14230 35176813 PMC9303595

[B6] Angeles-Lopez Q. D. Rodriguez-Lopez J. Agudelo Garcia P. Calyeca J. Álvarez D. Bueno M. (2025). Regulation of lung progenitor plasticity and repair by fatty acid oxidation. JCI Insight 10, e165837. 10.1172/jci.insight.165837 39927460 PMC11948574

[B7] Apte S. H. Lutzky V. P. Groves P. L. Chambers D. C. (2026). Alveolar lipid-macrophage networks at the intersection of pulmonary fibrosis. Cells 15, 668. 10.3390/cells15080668 42041536 PMC13115404

[B8] Bai X. Zhao G. Chen Q. Li Z. Gao M. Ho W. (2022). Inhaled siRNA nanoparticles targeting IL11 inhibit lung fibrosis and improve pulmonary function post-bleomycin challenge. Sci. Adv. 8, eabn7162. 10.1126/sciadv.abn7162 35731866 PMC9216512

[B9] Bai X. Chen Q. Li F. Teng Y. Tang M. Huang J. (2024). Optimized inhaled LNP formulation for enhanced treatment of idiopathic pulmonary fibrosis via mRNA-mediated antibody therapy. Nat. Commun. 15, 6844. 10.1038/s41467-024-51056-8 39122711 PMC11315999

[B10] Benador I. Y. Veliova M. Mahdaviani K. Petcherski A. Wikstrom J. D. Assali E. A. (2018). Mitochondria bound to lipid droplets have unique bioenergetics, composition, and dynamics that support lipid droplet expansion. Cell Metab. 27, 869–885.e6. 10.1016/j.cmet.2018.03.003 29617645 PMC5969538

[B11] Bezawork-Geleta A. Devereux C. J. Keenan S. N. Lou J. Cho E. Nie S. (2025). Proximity proteomics reveals a mechanism of fatty acid transfer at lipid droplet-mitochondria- endoplasmic reticulum contact sites. Nat. Commun. 16, 2135. 10.1038/s41467-025-57405-5 40032835 PMC11876333

[B12] Bonella F. Spagnolo P. Ryerson C. (2023). Current and future treatment landscape for idiopathic pulmonary fibrosis. Drugs 83, 1581–1593. 10.1007/s40265-023-01950-0 PMC10693523 37882943

[B13] Bórquez J. C. Díaz-Castro F. La Fuente F. P. Espinoza K. Figueroa A. M. Martínez-Ruíz I. (2024). Mitofusin-2 induced by exercise modifies lipid droplet-mitochondria communication, promoting fatty acid oxidation in male mice with NAFLD. Metabolism 152, 155765. 10.1016/j.metabol.2023.155765 38142958

[B14] Bueno M. Calyeca J. Rojas M. Mora A. L. (2020). Mitochondria dysfunction and metabolic reprogramming as drivers of idiopathic pulmonary fibrosis. Redox Biol. 33, 101509. 10.1016/j.redox.2020.101509 32234292 PMC7251240

[B15] Cai R. Lin H. Cheng Q. Mao Q. Zhang C. Tan Y. (2024). Construction of a novel lipid drop-mitochondria-associated genetic profile for predicting the survival and prognosis of lung adenocarcinoma. Discov. Oncol. 15, 668. 10.1007/s12672-024-01526-8 39551861 PMC11570572

[B16] Calì T. Bayer E. M. Eden E. R. Hajnóczky G. Kornmann B. Lackner L. (2025). Key challenges and recommendations for defining organelle membrane contact sites. Nat. Rev. Mol. Cell Biol. 26, 776–796. 10.1038/s41580-025-00864-x 40550870

[B17] Cañadas O. Olmeda B. Alonso A. Pérez-Gil J. (2020). Lipid-protein and protein-protein interactions in the pulmonary surfactant system and their role in lung homeostasis. Int. J. Mol. Sci. 21, 3708. 10.3390/ijms21103708 32466119 PMC7279303

[B18] Cantor J. (2025). Rodent models of lung disease: a road map for translational research. Int. J. Mol. Sci. 26, 8386. 10.3390/ijms26178386 40943307 PMC12429320

[B19] Cevallos C. Jarmoluk P. Sviercz F. Freiberger R. N. López C. A. M. Delpino M. V. (2025). Ferroptosis and mitochondrial ROS are central to SARS-CoV-2-induced hepatocyte death. Front. Cell Infect. Microbiol. 15, 1625928. 10.3389/fcimb.2025.1625928 40861496 PMC12375647

[B20] Chen X. Xiao C. Liu Y. Li Q. Cheng Y. Li S. (2023a). HUB genes transcriptionally regulate lipid metabolism in alveolar type II cells under LPS stimulation. Heliyon 9, e19437. 10.1016/j.heliyon.2023.e19437 PMC10472236 37662799

[B21] Chen P. Wu M. He Y. Jiang B. He M.-L. (2023b). Metabolic alterations upon SARS-CoV-2 infection and potential therapeutic targets against coronavirus infection. Signal Transduct. Target Ther. 8, 237. 10.1038/s41392-023-01510-8 PMC10244875 37286535

[B22] Chen Z. Kong X. Ma Q. Chen J. Zeng Y. Liu H. (2024). The impact of mycobacterium tuberculosis on the macrophage cholesterol metabolism pathway. Front. Immunol. 15, 1402024. 10.3389/fimmu.2024.1402024 38873598 PMC11169584

[B23] Chung K.-P. Hsu C.-L. Fan L.-C. Huang Z. Bhatia D. Chen Y.-J. (2019). Mitofusins regulate lipid metabolism to mediate the development of lung fibrosis. Nat. Commun. 10, 3390. 10.1038/s41467-019-11327-1 31358769 PMC6662701

[B24] Cloonan S. M. Kim K. Esteves P. Trian T. Barnes P. J. (2020). Mitochondrial dysfunction in lung ageing and disease. Eur. Respir. Rev. 29, 200165. 10.1183/16000617.0165-2020 33060165 PMC9488551

[B25] Cui L. Liu P. (2020). Two types of contact between lipid droplets and mitochondria. Front. Cell Dev. Biol. 8, 618322. 10.3389/fcell.2020.618322 33385001 PMC7769837

[B26] de Assis Fernandes Caldeira D. Dutra S. J. Martins M. M. Gonzaga Veras R. McAuley D. F. Rieken M. (2025). Transplantation of mesenchymal stromal cell-derived mitochondria alleviates endothelial dysfunction in pre-clinical models of acute respiratory distress syndrome. Stem Cells Transl. Med. 14, szaf053. 10.1093/stcltm/szaf053 PMC12618169 41239558

[B27] De Oliveira M. P. Liesa M. (2020). The role of mitochondrial fat oxidation in cancer cell proliferation and survival. Cells 9, 2600. 10.3390/cells9122600 33291682 PMC7761891

[B28] Deng X. Yang Y. Gan L. Duan X. Wang X. Zhang J. (2025). Engineering lipid nanoparticles to enhance intracellular delivery of transforming growth factor-beta siRNA (siTGF-β1) via inhalation for improving pulmonary fibrosis post-bleomycin challenge. Pharmaceutics 17, 157. 10.3390/pharmaceutics17020157 40006524 PMC11859093

[B29] Dias S. S. G. Soares V. C. Ferreira A. C. Sacramento C. Q. Fintelman-Rodrigues N. Temerozo J. R. (2020). Lipid droplets fuel SARS-CoV-2 replication and production of inflammatory mediators. PLoS Pathog. 16, e1009127. 10.1371/journal.ppat.1009127 33326472 PMC7773323

[B30] Dietl P. Frick M. (2021). Channels and transporters of the pulmonary lamellar body in health and disease. Cells 11, 45. 10.3390/cells11010045 35011607 PMC8750383

[B31] Ding F. Zhou M. Ren Y. Li Y. Xiang J. Li Y. (2024). Mitochondrial extracellular vesicles: a promising avenue for diagnosing and treating lung diseases. ACS Nano 18, 25372–25404. 10.1021/acsnano.4c02940 39225081

[B32] Diokmetzidou A. Scorrano L. (2025). A guide to characterizing the dynamic mitochondria-endoplasmic reticulum contact sites. FEBS J. 292, 6497–6511. 10.1111/febs.70184 PMC12713337 40622131

[B33] Dong Y. Arif A. A. Guo J. Ha Z. Lee-Sayer S. S. M. Poon G. F. T. (2020). CD44 loss disrupts lung lipid surfactant homeostasis and exacerbates oxidized lipid-induced lung inflammation. Front. Immunol. 11, 29. 10.3389/fimmu.2020.00029 32082314 PMC7002364

[B34] D’Avila H. Lima C. N. R. Rampinelli P. G. Mateus L. C. O. Sousa Silva R. V. de Correa J. R. (2024). Lipid metabolism modulation during SARS-CoV-2 infection: a spotlight on extracellular vesicles and therapeutic prospects. Int. J. Mol. Sci. 25, 640. 10.3390/ijms25010640 38203811 PMC10778989

[B35] Eltayeb K. La Monica S. Tiseo M. Alfieri R. Fumarola C. (2022). Reprogramming of lipid metabolism in lung cancer: an overview with focus on EGFR-mutated non-small cell lung cancer. Cells 11, 413. 10.3390/cells11030413 35159223 PMC8834094

[B36] Enkler L. Spang A. (2024). Functional interplay of lipid droplets and mitochondria. FEBS Lett. 598, 1235–1251. 10.1002/1873-3468.14809 38268392

[B37] Esteves P. Blanc L. Celle A. Dupin I. Maurat E. Amoedo N. (2021). Crucial role of fatty acid oxidation in asthmatic bronchial smooth muscle remodelling. Eur. Respir. J. 58, 2004252. 10.1183/13993003.04252-2020 33833033

[B38] Fairley L. H. Das S. Dharwal V. Amorim N. Hegarty K. J. Wadhwa R. (2023). Mitochondria-targeted antioxidants as a therapeutic strategy for chronic obstructive pulmonary disease. Antioxidants (Basel) 12, 973. 10.3390/antiox12040973 37107348 PMC10135688

[B39] Falahati M. Hasan A. Zeinabad H. A. Serpooshan V. von der Thüsen J. H. ten Hagen T. L. M. (2023). Engineering of pulmonary surfactant corona on inhaled nanoparticles to operate in the lung system. Nano Today 52, 101998. 10.1016/j.nantod.2023.101998

[B40] Fan H. Tan Y. (2024). Lipid droplet-mitochondria contacts in health and disease. Int. J. Mol. Sci. 25, 6878. 10.3390/ijms25136878 38999988 PMC11240910

[B41] Fan L.-C. McConn K. Plataki M. Kenny S. Williams N. C. Kim K. (2023). Alveolar type II epithelial cell FASN maintains lipid homeostasis in experimental COPD. JCI Insight 8, e163403. 10.1172/jci.insight.163403 37606038 PMC10543729

[B42] Flerlage T. Crawford J. C. Allen E. K. Severns D. Tan S. Surman S. (2023). Single cell transcriptomics identifies distinct profiles in pediatric acute respiratory distress syndrome. Nat. Commun. 14, 3870. 10.1038/s41467-023-39593-0 37391405 PMC10313703

[B43] Franzén L. Olsson L. M. Hühn M. Ptasinski V. Setyo L. Keith B. P. (2024). Mapping spatially resolved transcriptomes in human and mouse pulmonary fibrosis. Nat. Genet. 56, 1725–1736. 10.1038/s41588-024-01819-2 38951642 PMC11319205

[B44] Freyre C. A. C. Rauher P. C. Ejsing C. S. Klemm R. W. (2019). MIGA2 links mitochondria, the ER, and lipid droplets and promotes *de novo* lipogenesis in adipocytes. Mol. Cell 76, 811–825.e14. 10.1016/j.molcel.2019.09.011 31628041

[B45] From the American Association of Neurological Surgeons (AANS), American Society of Neuroradiology (ASNR), Cardiovascular and Interventional Radiology Society of Europe (CIRSE), Can, adian Interventional Radiology Association (CIRA), Congress of Neurological Surgeons (CNS), European Society of Minimally Invasive Neurological Therapy (ESMINT), European Society of Neuroradiology (ESNR), European Stroke Organization (ESO), Society for Car, diovascular Angiography and Interventions (SCAI), Society of Interventional Radiology (SIR), Society of NeuroInterventional Surgery (SNIS), and World Stroke Organization (WSO), Sacks D. Baxter B. Campbell B. C. V. Carpenter J. S. Cognard C. (2018). Multisociety consensus quality improvement revised consensus statement for endovascular therapy of acute ischemic stroke. Int. J. Stroke 13, 612–632. 10.1177/1747493018778713 29786478

[B46] Geng J. Liu Y. Dai H. Wang C. (2021). Fatty acid metabolism and idiopathic pulmonary fibrosis. Front. Physiol. 12, 794629. 10.3389/fphys.2021.794629 35095559 PMC8795701

[B47] Genoula M. Marín Franco J. L. Maio M. Dolotowicz B. Ferreyra M. Milillo M. A. (2020). Fatty acid oxidation of alternatively activated macrophages prevents foam cell formation, but mycobacterium tuberculosis counteracts this process via HIF-1α activation. PLoS Pathog. 16, e1008929. 10.1371/journal.ppat.1008929 33002063 PMC7553279

[B48] Giambelluca S. Ochs M. Lopez-Rodriguez E. (2025). Cholesterol-mediated inflammation activation in alveolar macrophages. BMC Biol. 23, 369. 10.1186/s12915-025-02494-3 41430235 PMC12750553

[B49] Gonsalves A. Menon J. U. (2024). Impact of nebulization on the physicochemical properties of polymer-lipid hybrid nanoparticles for pulmonary drug delivery. Int. J. Mol. Sci. 25, 5028. 10.3390/ijms25095028 38732246 PMC11084240

[B50] Griseti E. Bello A. A. Bieth E. Sabbagh B. Iacovoni J. S. Bigay J. (2024). Molecular mechanisms of perilipin protein function in lipid droplet metabolism. FEBS Lett. 598, 1170–1198. 10.1002/1873-3468.14792 38140813

[B51] Gründing A. R. Schneider M. A. Richtmann S. Kriegsmann M. Winter H. Martinez-Delgado B. (2022). Lung adenocarcinoma cell sensitivity to chemotherapies: a spotlight on lipid droplets and SREBF1 gene. Cancers (Basel) 14, 4454. 10.3390/cancers14184454 36139614 PMC9497419

[B52] Gu L. Surolia R. Larson-Casey J. L. He C. Davis D. Kang J. (2022). Targeting Cpt1a-bcl-2 interaction modulates apoptosis resistance and fibrotic remodeling. Cell Death Differ. 29, 118–132. 10.1038/s41418-021-00840-w 34413485 PMC8738732

[B53] Guerrini V. Panettieri R. A. Gennaro M. L. (2020). Lipid-laden macrophages as biomarkers of vaping-associated lung injury. Lancet Respir. Med. 8, e6. 10.1016/S2213-2600(19)30476-X 32035068 PMC7297059

[B54] Haeger S. Moore C. M. McManus S. A. Moore P. K. Janssen W. J. Mould K. J. (2024). The bronchoalveolar lavage dilution conundrum: an updated view on a long-standing problem. Am. J. Physiol. Lung Cell Mol. Physiol. 327, L807–L813. 10.1152/ajplung.00054.2024 39378107 PMC11560068

[B55] Han M. Bushong E. A. Segawa M. Tiard A. Wong A. Brady M. R. (2023). Spatial mapping of mitochondrial networks and bioenergetics in lung cancer. Nature 615, 712–719. 10.1038/s41586-023-05793-3 36922590 PMC10033418

[B56] Hao Z. Wang H. Zhou Z. Yang Q. Zhang B. Ma J. (2024). HPS6 deficiency leads to reduced vacuolar-type H+-ATPase and impaired biogenesis of lamellar bodies in alveolar type II cells. Am. J. Respir. Cell Mol. Biol. 71, 442–452. 10.1165/rcmb.2022-0492OC 38864759

[B57] Hartsoe P. Holguin F. Chu H. W. (2024). Mitochondrial dysfunction and metabolic reprogramming in obesity and asthma. Int. J. Mol. Sci. 25, 2944. 10.3390/ijms25052944 38474191 PMC10931700

[B58] Herker E. Vieyres G. Beller M. Krahmer N. Bohnert M. (2021). Lipid droplet contact sites in health and disease. Trends Cell Biol. 31, 345–358. 10.1016/j.tcb.2021.01.004 33546922

[B59] Hernández-Hernández I. De La Rosa J. V. Martín-Rodríguez P. Díaz-Sarmiento M. Recio C. Guerra B. (2024). Endogenous LXR signaling controls pulmonary surfactant homeostasis and prevents lung inflammation. Cell Mol. Life Sci. 81, 287. 10.1007/s00018-024-05310-3 38970705 PMC11335212

[B60] Hou Y. Wei D. Zhang Z. Guo H. Li S. Zhang J. (2022). FABP5 controls macrophage alternative activation and allergic asthma by selectively programming long-chain unsaturated fatty acid metabolism. Cell Rep. 41, 111668. 10.1016/j.celrep.2022.111668 36384126

[B61] Huang C. Du W. Ni Y. Lan G. Shi G. (2022). The effect of short-chain fatty acids on M2 macrophages polarization *in vitro* and *in vivo* . Clin. Exp. Immunol. 207, 53–64. 10.1093/cei/uxab028 35020860 PMC8802183

[B62] Jin C. Yuan P. (2020). Implications of lipid droplets in lung cancer: associations with drug resistance. Oncol. Lett. 20, 2091–2104. 10.3892/ol.2020.11769 32782526 PMC7399769

[B63] K K. S. P. Narayansamy D. (2025). Advancements in nanotechnology for targeted drug delivery in idiopathic pulmonary fibrosis: a focus on solid lipid nanoparticles and nanostructured lipid carriers. Drug Dev. Ind. Pharm. 51, 285–294. 10.1080/03639045.2025.2468811 39963904

[B64] Kai F. Leidal A. M. Weaver V. M. (2025). Tension-induced organelle stress: an emerging target in fibrosis. Trends Pharmacol. Sci. 46, 117–131. 10.1016/j.tips.2024.12.006 39818520 PMC11805623

[B65] Kalam H. Chou C.-H. Kadoki M. Graham D. B. Deguine J. Hung D. T. (2023). Identification of host regulators of mycobacterium tuberculosis phenotypes uncovers a role for the MMGT1-GPR156 lipid droplet axis in persistence. Cell Host Microbe 31, 978–992.e5. 10.1016/j.chom.2023.05.009 37269834 PMC10373099

[B66] Kallinos E. Chung K.-P. Torres L. K. Bhatia D. Ersoy B. Carmeliet P. (2025). High-fat diet obesity exacerbates acute lung injury-induced dysregulation of fatty acid oxidation in alveolar epithelial type 2 cells. Am. J. Physiol. Lung Cell Mol. Physiol. 329, L343–L356. 10.1152/ajplung.00406.2024 40695588 PMC12352525

[B67] Kang S. W. S. Brown L. A. Miller C. B. Barrows K. M. Golino J. L. Liu H. (2026). PLIN5 phosphorylation orchestrates mitochondria lipid-droplet coupling to control hepatic lipid flux and steatosis. Nat. Metab. 8, 587–603. 10.1038/s42255-026-01476-1 41872512 PMC13031124

[B68] Karampitsakos T. Juan-Guardela B. M. Tzouvelekis A. Herazo-Maya J. D. (2023). Precision medicine advances in idiopathic pulmonary fibrosis. EBioMedicine 95, 104766. 10.1016/j.ebiom.2023.104766 37625268 PMC10469771

[B69] Kassab G. Doran K. Mo Y. Zheng G. (2023). Inhalable gene therapy and the lung surfactant problem. Nano Lett. 23, 10099–10102. 10.1021/acs.nanolett.3c03547 37930273

[B70] Kawano-Dourado L. Kulkarni T. Ryerson C. J. Rivera-Ortega P. Baldi B. G. Chaudhuri N. (2024). Adaptive multi-interventional trial platform to improve patient care for fibrotic interstitial lung diseases. Thorax 79, 788–795. 10.1136/thorax-2023-221148 38448221 PMC11287572

[B71] Kien B. Kolleritsch S. Kunowska N. Heier C. Chalhoub G. Tilp A. (2022). Lipid droplet-mitochondria coupling via perilipin 5 augments respiratory capacity but is dispensable for FA oxidation. J. Lipid Res. 63, 100172. 10.1016/j.jlr.2022.100172 PMC8953689 35065923

[B72] Kim H. Lee S. Jun Y. Lee C. (2022). Structural basis for mitoguardin-2 mediated lipid transport at ER-mitochondrial membrane contact sites. Nat. Commun. 13, 3702. 10.1038/s41467-022-31462-6 35764626 PMC9239997

[B73] Klein S. Wimmer B. H. Winter S. L. Kolovou A. Laketa V. Chlanda P. (2021). Post-correlation on-lamella cryo-CLEM reveals the membrane architecture of lamellar bodies. Commun. Biol. 4, 137. 10.1038/s42003-020-01567-z 33514845 PMC7846596

[B74] Knight M. Braverman J. Asfaha K. Gronert K. Stanley S. (2018). Lipid droplet formation in mycobacterium tuberculosis infected macrophages requires IFN-γ/HIF-1α signaling and supports host defense. PLoS Pathog. 14, e1006874. 10.1371/journal.ppat.1006874 29370315 PMC5800697

[B75] Ko M. You J. Cho E. Kwon H. J. (2026). Spatial pharmacology: redefining organelle contact sites as therapeutic targets. Int. J. Biol. Sci. 22, 2121–2123. 10.7150/ijbs.129515 41694591 PMC12905643

[B76] Kook S. Wang P. Meng S. Jetter C. S. Sucre J. M. S. Benjamin J. T. (2021). AP-3-dependent targeting of flippase ATP8A1 to lamellar bodies suppresses activation of YAP in alveolar epithelial type 2 cells. Proc. Natl. Acad. Sci. U. S. A. 118, e2025208118. 10.1073/pnas.2025208118 33990468 PMC8157957

[B77] Kumari S. Singh K. Khadia M. Kumar R. Bansal V. Mishra A. (2025). Exploring the influence of metabolic changes in fibrotic lung diseases. Pulm. Circ. 15, e70163. 10.1002/pul2.70163 40989087 PMC12452044

[B78] Lancaster N. M. Sinitcyn P. Forny P. Peters-Clarke T. M. Fecher C. Smith A. J. (2024). Fast and deep phosphoproteome analysis with the orbitrap astral mass spectrometer. Nat. Commun. 15, 7016. 10.1038/s41467-024-51274-0 39147754 PMC11327265

[B79] Larson-Casey J. L. He C. Carter A. B. (2020). Mitochondrial quality control in pulmonary fibrosis. Redox Biol. 33, 101426. 10.1016/j.redox.2020.101426 31928788 PMC7251238

[B80] Laval T. Chaumont L. Demangel C. (2021). Not too fat to fight: the emerging role of macrophage fatty acid metabolism in immunity to mycobacterium tuberculosis. Immunol. Rev. 301, 84–97. 10.1111/imr.12952 33559209

[B81] Lee M. H. Sanders L. Kumar R. Hernandez-Saavedra D. Yun X. Ford J. A. (2022). Contribution of fatty acid oxidation to the pathogenesis of pulmonary hypertension. Am. J. Physiol. Lung Cell Mol. Physiol. 323, L355–L371. 10.1152/ajplung.00039.2022 35763400 PMC9448289

[B82] Lee E. Williams K. J. McCarthy C. Bridges J. P. Redente E. F. de Aguiar Vallim T. Q. (2024a). Alveolar macrophage lipid burden correlates with clinical improvement in patients with pulmonary alveolar proteinosis. J. Lipid Res. 65, 100496. 10.1016/j.jlr.2024.100496 38185217 PMC10844116

[B83] Lee S.-E. Kim I.-H. Kang Y. C. Kim Y. Yu S.-H. Yeo J. S. (2024b). Mitochondrial transplantation attenuates lipopolysaccharide-induced acute respiratory distress syndrome. BMC Pulm. Med. 24, 477. 10.1186/s12890-024-03304-2 39334020 PMC11437886

[B84] Lee S.-H. Kim K. Lee E. Lee K. Ahn K. H. Park H. (2024c). Prediction of TKI response in EGFR-mutant lung cancer patients-derived organoids using malignant pleural effusion. NPJ Precis. Oncol. 8, 111. 10.1038/s41698-024-00609-7 PMC11109121 38773241

[B85] Li X. Wang L. Hao J. Zhu Q. Guo M. Wu C. (2022). The role of autophagy in lamellar body formation and surfactant production in type 2 alveolar epithelial cells. Int. J. Biol. Sci. 18, 1107–1119. 10.7150/ijbs.64285 35173542 PMC8771840

[B86] Li D. Zhao A. Zhu J. Wang C. Shen J. Zheng Z. (2023a). Inhaled lipid nanoparticles alleviate established pulmonary fibrosis. Small 19, e2300545. 10.1002/smll.202300545 37058092

[B87] Li J. Xiao Y. Zhang Y. Li S. Zhao M. Xia T. (2023b). Pulmonary delivery of specialized pro-resolving mediators-based nanotherapeutics attenuates pulmonary fibrosis in preclinical animal models. ACS Nano 17, 15354–15370. 10.1021/acsnano.2c10388 37535431

[B88] Li Z. Hui X. Zheng M. He Z. Huai W. Ding B. (2025a). Reprogramming of lipid metabolism by ORF3a-induced microlipophagy enhances biogenesis of SARS-CoV-2 replication organelle. PLoS Pathog. 21, e1013676. 10.1371/journal.ppat.1013676 41270099 PMC12671888

[B89] Li W. Xie Y. Chen Z. Cao D. Wang Y. (2025b). Epithelial-mesenchymal transition in pulmonary fibrosis: molecular mechanisms and emerging therapeutic strategies. Front. Med. (Lausanne) 12, 1658001. 10.3389/fmed.2025.1658001 40988754 PMC12450681

[B90] Little I. Bersie S. Redente E. F. McCubbrey A. L. Tarling E. J. (2025). Alveolar macrophages: guardians of the alveolar lipid galaxy. Curr. Opin. Lipidol. 36, 153–162. 10.1097/MOL.0000000000000987 40183504 PMC12043416

[B91] Liu J. Wu Z. Liu Y. Zhan Z. Yang L. Wang C. (2022). ROS-responsive liposomes as an inhaled drug delivery nanoplatform for idiopathic pulmonary fibrosis treatment via Nrf2 signaling. J. Nanobiotechnology 20, 213. 10.1186/s12951-022-01435-4 35524280 PMC9074278

[B92] Liu Y. Sun Y. Guo Y. Shi X. Chen X. Feng W. (2023). An overview: the diversified role of mitochondria in cancer metabolism. Int. J. Biol. Sci. 19, 897–915. 10.7150/ijbs.81609 36778129 PMC9910000

[B93] López-Ayllón B. D. Marin S. Fernández M. F. García-García T. Fernández-Rodríguez R. de Lucas-Rius A. (2024). Metabolic and mitochondria alterations induced by SARS-CoV-2 accessory proteins ORF3a, ORF9b, ORF9c and ORF10. J. Med. Virol. 96, e29752. 10.1002/jmv.29752 38949191

[B94] Lu A. Xu Z. Zhao Z. Yan Y. Jiang L. Geng J. (2024). Double braking effects of nanomedicine on mitochondrial permeability transition pore for treating idiopathic pulmonary fibrosis. Adv. Sci. (Weinh) 11, e2405406. 10.1002/advs.202405406 39475000 PMC11653616

[B95] Lu L. Shi M. Qin W. Yang M. Wang X. Wang Y. (2025). Immunometabolic programming of macrophages in asthma pathogenesis and therapy. Front. Physiol. 16, 1736340. 10.3389/fphys.2025.1736340 41602451 PMC12832530

[B96] Lung J. Hung M.-S. Wang T.-Y. Chen K.-L. Luo C.-W. Jiang Y.-Y. (2022). Lipid droplets in lung cancers are crucial for the cell growth and starvation survival. Int. J. Mol. Sci. 23, 12533. 10.3390/ijms232012533 36293388 PMC9604479

[B97] Ma L. Chen C. Zhao C. Li T. Ma L. Jiang J. (2024). Targeting carnitine palmitoyl transferase 1A (CPT1A) induces ferroptosis and synergizes with immunotherapy in lung cancer. Signal Transduct. Target Ther. 9, 64. 10.1038/s41392-024-01772-w PMC10920667 38453925

[B98] MacIsaac S. Somboonviboon D. Scallan C. Kolb M. (2024). Treatment of idiopathic pulmonary fibrosis: an update on emerging drugs in phase II and III clinical trials. Expert Opin. Emerg. Drugs 29, 177–186. 10.1080/14728214.2024.2340723 38588523

[B99] Manevski M. Muthumalage T. Devadoss D. Sundar I. K. Wang Q. Singh K. P. (2020). Cellular stress responses and dysfunctional mitochondrial-cellular senescence, and therapeutics in chronic respiratory diseases. Redox Biol. 33, 101443. 10.1016/j.redox.2020.101443 32037306 PMC7251248

[B100] Marshall A. G. Damo S. M. Hinton A. (2023). Revisiting focused ion beam scanning electron microscopy. Trends Biochem. Sci. 48, 585–586. 10.1016/j.tibs.2023.02.005 36990957

[B101] Mathiowetz A. J. Olzmann J. A. (2024). Lipid droplets and cellular lipid flux. Nat. Cell Biol. 26, 331–345. 10.1038/s41556-024-01364-4 38454048 PMC11228001

[B102] Mawer C. M. A. Stanel S. C. Ward E. M. Smith D. J. F. Hull R. C. Mehta P. (2026). A framework for translational research in interstitial lung disease (ILD) using bronchoalveolar lavage (BAL). Expert Rev. Clin. Immunol. 22, 499–517. 10.1080/1744666X.2026.2675640 42136372

[B103] McClintock C. R. Mulholland N. Krasnodembskaya A. D. (2022). Biomarkers of mitochondrial dysfunction in acute respiratory distress syndrome: a systematic review and meta-analysis. Front. Med. (Lausanne) 9, 1011819. 10.3389/fmed.2022.1011819 36590959 PMC9795057

[B104] Mekonnen D. Derbie A. Mihret A. Yimer S. A. Tønjum T. Gelaw B. (2021). Lipid droplets and the transcriptome of mycobacterium tuberculosis from direct sputa: a literature review. Lipids Health Dis. 20, 129. 10.1186/s12944-021-01550-5 34602073 PMC8487580

[B105] Meng Y. Guo D. Lin L. Zhao H. Xu W. Luo S. (2024). Glycolytic enzyme PFKL governs lipolysis by promoting lipid droplet-mitochondria tethering to enhance β-oxidation and tumor cell proliferation. Nat. Metab. 6, 1092–1107. 10.1038/s42255-024-01047-2 38773347

[B106] Miner G. E. So C. M. Edwards W. Ragusa J. V. Wine J. T. Wong Gutierrez D. (2023). PLIN5 interacts with FATP4 at membrane contact sites to promote lipid droplet-to-mitochondria fatty acid transport. Dev. Cell 58, 1250–1265.e6. 10.1016/j.devcel.2023.05.006 37290445 PMC10525032

[B107] Miyata-Morita K. Kawashima A. Kiriya M. Dejima H. Saito K. Sakao Y. (2025). Perilipin 2 mediates progression of lung adenocarcinoma by modulating lipid metabolism. Am. J. Pathol. 195, 1588–1599. 10.1016/j.ajpath.2025.05.016 40541711 PMC12489354

[B108] Mohareer K. Medikonda J. Vadankula G. R. Banerjee S. (2020). Mycobacterial control of host mitochondria: bioenergetic and metabolic changes shaping cell fate and infection outcome. Front. Cell Infect. Microbiol. 10, 457. 10.3389/fcimb.2020.00457 33102245 PMC7554303

[B109] Morán-Lalangui M. Villoslada-González M. Hevia-Lorenzo C. Pérez-Gil J. García-Álvarez B. (2025). Pulmonary surfactant protein SP-C regulates lipid vesicle uptake by alveolar type II cells and macrophages: role of lipids, palmitoylation, and environment. Biochim. Biophys. Acta Mol. Cell Biol. Lipids 1870, 159688. 10.1016/j.bbalip.2025.159688 40925447

[B110] Muneer G. Chen C.-S. Chen Y.-J. (2025). Advancements in global phosphoproteomics profiling: overcoming challenges in sensitivity and quantification. Proteomics 25, e202400087. 10.1002/pmic.202400087 39696887 PMC11735659

[B111] Najt C. P. Khan S. A. Heden T. D. Witthuhn B. A. Perez M. Heier J. L. (2020). Lipid droplet-derived monounsaturated fatty acids traffic via PLIN5 to allosterically activate SIRT1. Mol. Cell 77, 810–824.e8. 10.1016/j.molcel.2019.12.003 31901447 PMC7036014

[B112] Nakamura Y. Shimizu Y. (2023). Cellular and molecular control of lipid metabolism in idiopathic pulmonary fibrosis: clinical application of the lysophosphatidic acid pathway. Cells 12, 548. 10.3390/cells12040548 36831215 PMC9954511

[B113] Nardacci R. Colavita F. Castilletti C. Lapa D. Matusali G. Meschi S. (2021). Evidences for lipid involvement in SARS-CoV-2 cytopathogenesis. Cell Death Dis. 12, 263. 10.1038/s41419-021-03527-9 33712574 PMC7952828

[B114] Ni F.-X. Hu J. Chen P.-S. Chen H.-H. Huang D.-H. Jiang Z.-B. (2025). Ferroptosis-immune-metabolic axis in asthma: mechanistic crosstalk, endotype-specific regulation, and translational targeting. Front. Immunol. 16, 1714608. 10.3389/fimmu.2025.1714608 41573584 PMC12819738

[B115] Ni F.-X. Chen H.-H. Jiang Z.-B. Huang D.-H. (2026). Ironing out COPD: Ferroptosis-driven immune dysregulation, metabolic rewiring, and precision therapeutic opportunities. Front. Immunol. 17, 1630969. 10.3389/fimmu.2026.1630969 41836443 PMC12982077

[B116] Nie Y. Li J. Zhai X. Wang Z. Wang J. Wu Y. (2023). Elamipretide(SS-31) attenuates idiopathic pulmonary fibrosis by inhibiting the Nrf2-dependent NLRP3 inflammasome in macrophages. Antioxidants (Basel) 12, 2022. 10.3390/antiox12122022 38136142 PMC10740969

[B117] Niu Y.-J. Liu J.-J. Zhang J.-H. Lu S.-Q. Wang K. Zhu Z. (2025). Perilipins: key targets for regulating lipid metabolism and alleviating abnormal lipid metabolism through exercise. Diabetol. Metab. Syndr. 17, 392. 10.1186/s13098-025-01965-5 41094515 PMC12522897

[B118] Nukala S. B. Tura-Ceide O. Aldini G. Smolders VFED Blanco I. Peinado V. I. (2021). Protein network analyses of pulmonary endothelial cells in chronic thromboembolic pulmonary hypertension. Sci. Rep. 11, 5583. 10.1038/s41598-021-85004-z PMC7946953 33692478

[B119] O’Connor R. S. Guo L. Ghassemi S. Snyder N. W. Worth A. J. Weng L. (2018). The CPT1a inhibitor, etomoxir induces severe oxidative stress at commonly used concentrations. Sci. Rep. 8, 6289. 10.1038/s41598-018-24676-6 PMC5908836 29674640

[B120] Park W. H. (2025). The mitochondrial nexus: dysfunction, inhibition, and therapeutic frontiers in lung disease. Respir. Med. 250, 108506. 10.1016/j.rmed.2025.108506 41241149

[B121] Peng Q. Liang W. Wang W. Wang J. Wang Q. Zhao C. (2026). Lipid droplet-mitochondria tethering releases MAVS inhibition to potentiate antiviral immunity. Cell Rep. 45, 117601. 10.1016/j.celrep.2026.117601 42329764

[B122] Pokharel M. D. Marciano D. P. Fu P. Franco M. C. Unwalla H. Tieu K. (2023). Metabolic reprogramming, oxidative stress, and pulmonary hypertension. Redox Biol. 64, 102797. 10.1016/j.redox.2023.102797 37392518 PMC10363484

[B123] Pokharel M. D. Garcia-Flores A. Marciano D. Franco M. C. Fineman J. R. Aggarwal S. (2024). Mitochondrial network dynamics in pulmonary disease: bridging the gap between inflammation, oxidative stress, and bioenergetics. Redox Biol. 70, 103049. 10.1016/j.redox.2024.103049 38295575 PMC10844980

[B124] Pranty A. I. Cuevas Ocaña S. Dickens J. A. (2026). Mechanisms of surfactant maturation in development and disease. Physiol. (Bethesda) 41, 0–380. 10.1152/physiol.00033.2025 41364119

[B125] Ran X. Yang Z. Cheng L. Li W. Cui J. Huang P. (2026). Multi-omics reveals cholesterol-driven macrophage metabolic reprogramming and inflammation in chronic obstructive pulmonary disease. Genome Med. 18, 17. 10.1186/s13073-025-01591-w 41486269 PMC12870984

[B126] Ravi A. Goorsenberg A. W. M. Dijkhuis A. Dierdorp B. S. Dekker T. van Weeghel M. (2021). Metabolic differences between bronchial epithelium from healthy individuals and patients with asthma and the effect of bronchial thermoplasty. J. Allergy Clin. Immunol. 148, 1236–1248. 10.1016/j.jaci.2020.12.653 33556463

[B127] Robinson M. J. Krasnodembskaya A. D. (2020). Therapeutic targeting of metabolic alterations in acute respiratory distress syndrome. Eur. Respir. Rev. 29, 200114. 10.1183/16000617.0114-2020 32620587 PMC9488567

[B128] Scur M. Mahmoud A. B. Dey S. Abdalbarri F. Stylianides I. Medina-Luna D. (2022). Alveolar macrophage metabolic programming via a C-type lectin receptor protects against lipo-toxicity and cell death. Nat. Commun. 13, 7272. 10.1038/s41467-022-34935-w 36433992 PMC9700784

[B129] Segalés J. Liesa M. (2026). The expanding role of mitochondria-lipid droplet contacts in liver and their disruption by MASLD. Nat. Metab. 8, 527–528. 10.1038/s42255-026-01483-2 41872513

[B130] Sever N. Miličić G. Bodnar N. O. Wu X. Rapoport T. A. (2021). Mechanism of lamellar body formation by lung surfactant protein B. Mol. Cell 81, 49–66.e8. 10.1016/j.molcel.2020.10.042 33242393 PMC7797001

[B131] Shen R. Qi Z. Huang X. Xia J. Zhan Q. (2024). Causal relationship between lipidome and acute respiratory distress syndrome. Sci. Rep. 14, 29523. 10.1038/s41598-024-80985-z 39604464 PMC11603036

[B132] Shi X. Chen Y. Shi M. Gao F. Huang L. Wang W. (2024). The novel molecular mechanism of pulmonary fibrosis: insight into lipid metabolism from reanalysis of single-cell RNA-seq databases. Lipids Health Dis. 23, 98. 10.1186/s12944-024-02062-8 38570797 PMC10988923

[B133] Shi G. Wang R. Huang C. (2025). Augmenting macrophages apoptosis induced by carnitine palmitoyl transferase 1A inhibition via acetyl-coa-associated protein acetylation. Immunology 175, 214–225. 10.1111/imm.13917 40071507

[B134] Shim D. Kim H. Shin S. J. (2020). Mycobacterium tuberculosis infection-driven foamy macrophages and their implications in tuberculosis control as targets for host-directed therapy. Front. Immunol. 11, 910. 10.3389/fimmu.2020.00910 32477367 PMC7235167

[B135] Siekacz K. Piotrowski W. J. Iwański M. A. Górski P. Białas A. J. (2021). The role of interaction between mitochondria and the extracellular matrix in the development of idiopathic pulmonary fibrosis. Oxid. Med. Cell Longev. 2021, 9932442. 10.1155/2021/9932442 34707784 PMC8545566

[B136] Smolková K. Gotvaldová K. (2025). Fatty acid trafficking between lipid droplets and mitochondria: an emerging perspective. Int. J. Biol. Sci. 21, 1863–1873. 10.7150/ijbs.105361 40083687 PMC11900811

[B137] Stephens I. O. Johnson L. A. (2025). Knockout of perilipin-2 in microglia alters lipid droplet accumulation and response to alzheimer’s disease stimuli. Cells 14, 1783. 10.3390/cells14221783 41294836 PMC12651832

[B138] Stevenson E. R. Smith L. C. Wilkinson M. L. Lee S. J. Gow A. J. (2023). Etiology of lipid-laden macrophages in the lung. Int. Immunopharmacol. 123, 110719. 10.1016/j.intimp.2023.110719 37595492 PMC10734282

[B139] Sun Y. L. Hennessey E. E. Heins H. Yang P. Villacorta-Martin C. Kwan J. (2024). Human pluripotent stem cell modeling of alveolar type 2 cell dysfunction caused by ABCA3 mutations. J. Clin. Invest 134, e164274. 10.1172/JCI164274 38226623 PMC10786693

[B140] Talari N. K. Mattam U. Meher N. K. Paripati A. K. Mahadev K. Krishnamoorthy T. (2023). Lipid-droplet associated mitochondria promote fatty-acid oxidation through a distinct bioenergetic pattern in male wistar rats. Nat. Commun. 14, 766. 10.1038/s41467-023-36432-0 36765117 PMC9918515

[B141] Tian Y. Duan C. Feng J. Liao J. Yang Y. Sun W. (2023). Roles of lipid metabolism and its regulatory mechanism in idiopathic pulmonary fibrosis: a review. Int. J. Biochem. Cell Biol. 155, 106361. 10.1016/j.biocel.2022.106361 36592687

[B142] Torresano L. Santacatterina F. Domínguez-Zorita S. Nuevo-Tapioles C. Núñez-Salgado A. Esparza-Moltó P. B. (2022). Analysis of the metabolic proteome of lung adenocarcinomas by reverse-phase protein arrays (RPPA) emphasizes mitochondria as targets for therapy. Oncogenesis 11, 24. 10.1038/s41389-022-00400-y PMC9085865 35534478

[B143] Wan Q. Zhang X. Zhou D. Xie R. Cai Y. Zhang K. (2023). Inhaled nano-based therapeutics for pulmonary fibrosis: recent advances and future prospects. J. Nanobiotechnology 21, 215. 10.1186/s12951-023-01971-7 37422665 PMC10329312

[B144] Wang H. Wen X. Yan M. Chang M. Zhang G. Peng W. (2020). The role of perilipin 2 in pseudomonas aeruginosa pulmonary infection. Respir. Physiol. Neurobiol. 281, 103497. 10.1016/j.resp.2020.103497 32683072

[B145] Wang W. Qu Y. Wang X. Xiao M. Z. X. Fu J. Chen L. (2023). Genetic variety of ORF3a shapes SARS-CoV-2 fitness through modulation of lipid droplet. J. Med. Virol. 95, e28630. 10.1002/jmv.28630 36861654

[B146] Wang Y. Zhang J. Liu Y. Yue X. Han K. Kong Z. (2024a). Realveolarization with inhalable mucus-penetrating lipid nanoparticles for the treatment of pulmonary fibrosis in mice. Sci. Adv. 10, eado4791. 10.1126/sciadv.ado4791 PMC11168475 38865465

[B147] Wang G.-Y. Xu X. Xiong D.-Y. Deng L. Liu W. Huang X.-T. (2024b). CPT1A as a potential therapeutic target for lipopolysaccharide-induced acute lung injury in mice. Sci. Rep. 14, 1600. 10.1038/s41598-024-52042-2 38238472 PMC10796431

[B148] Wang Y. Hu L.-F. Liu N.-H. Yang J.-S. Xing L. Jeong J.-H. (2024c). Mitophagy-enhanced nanoparticle-engineered mitochondria restore homeostasis of mitochondrial pool for alleviating pulmonary fibrosis. ACS Nano 18, 32705–32722. 10.1021/acsnano.4c10328 39546755

[B149] Wang M. Wu D. Liao X. Hu H. Gao J. Meng L. (2024d). CPT1A-IL-10-mediated macrophage metabolic and phenotypic alterations ameliorate acute lung injury. Clin. Transl. Med. 14, e1785. 10.1002/ctm2.1785 39090662 PMC11294017

[B150] Wang G. Sun B. Liu H. Hu M. Xu H. Li H. (2025a). Interactions between lipid droplets and mitochondria in metabolic diseases. Lipids Health Dis. 24, 357. 10.1186/s12944-025-02759-4 PMC12607196 41219930

[B151] Wang J. Meng S. Chen Y. Wang H. Hu W. Liu S. (2025b). MSC-mediated mitochondrial transfer promotes metabolic reprograming in endothelial cells and vascular regeneration in ARDS. Redox Rep. 30, 2474897. 10.1080/13510002.2025.2474897 PMC11912292 40082392

[B152] Wang F. Ge R. Cai Y. Zhao M. Fang Z. Li J. (2025c). Oxidative stress in ARDS: mechanisms and therapeutic potential. Front. Pharmacol. 16, 1603287. 10.3389/fphar.2025.1603287 40642004 PMC12241040

[B153] Wang M. He Y. Hu H. Wu D. Liao X. Gao J. (2026). Protective role of fatty acid oxidation against epithelial barrier dysfunction in allergic asthma. Redox Rep. 31, 2613534. 10.1080/13510002.2026.2613534 41555513 PMC12821354

[B154] Wu C. Dai C. Li X. Sun M. Chu H. Xuan Q. (2022). AKR1C3-dependent lipid droplet formation confers hepatocellular carcinoma cell adaptability to targeted therapy. Theranostics 12, 7681–7698. 10.7150/thno.74974 36451864 PMC9706585

[B155] Wu Y. Zheng K. Dong L. Gao S. Wu Y. Liu Z. (2026). SREBP2 promotes macrophage alternative activation and allergic airway inflammation independent of cholesterol biosynthesis. Cell Death Dis. 17, 697. 10.1038/s41419-026-08910-y PMC13448065 42236481

[B156] Xie C. Yalikun M. Ruan Z. Yu H. Huang X. Zhu H. (2025). Macrophage immunometabolism in pulmonary homeostasis and chronic lung diseases. Int. J. Biol. Sci. 21, 6580–6598. 10.7150/ijbs.123492 41281740 PMC12631069

[B157] Xu W. Ma X. Wang Q. Ye J. Wang N. Ye Z. (2023). GCN5L1 regulates pulmonary surfactant production by modulating lamellar body biogenesis and trafficking in mouse alveolar epithelial cells. Cell Mol. Biol. Lett. 28, 90. 10.1186/s11658-023-00506-0 37936104 PMC10631113

[B158] Xu D. Liu B. Wang L. (2025). MiR-365-3p inhibits lung cancer proliferation and migration via CPT1A-mediated fatty acid oxidation. Sci. Rep. 15, 7076. 10.1038/s41598-025-91665-x PMC11868381 40016582

[B159] Yang J. Pan X. Xu M. Li Y. Liang C. Liu L. (2024). Downregulation of HMGCS2 mediated AECIIs lipid metabolic alteration promotes pulmonary fibrosis by activating fibroblasts. Respir. Res. 25, 176. 10.1186/s12931-024-02816-z 38658970 PMC11040761

[B160] Yang S. Liu H. Zheng Y. Chu H. Lu Z. Yuan J. (2025). The role of PLIN3 in prognosis and tumor-associated macrophage infiltration: a pan-cancer analysis. J. Inflamm. Res. 18, 3757–3777. 10.2147/JIR.S509245 40098998 PMC11913039

[B161] Yao C.-H. Liu G.-Y. Wang R. Moon S. H. Gross R. W. Patti G. J. (2018). Identifying off-target effects of etomoxir reveals that carnitine palmitoyltransferase I is essential for cancer cell proliferation independent of β-oxidation. PLoS Biol. 16, e2003782. 10.1371/journal.pbio.2003782 29596410 PMC5892939

[B162] Yao X. Wen Z. Liao J. Meng Y. Lu P. Li X. (2026). DPEP1 mediates regulation of mitochondrial quality control via FOXO1/ALDH1L2 axis to attenuate ferroptosis in pulmonary endothelial cells to alleviate sepsis-associated acute lung injury. Int. Immunopharmacol. 170, 116049. 10.1016/j.intimp.2025.116049 41421227

[B163] Zadoorian A. Du X. Yang H. (2023). Lipid droplet biogenesis and functions in health and disease. Nat. Rev. Endocrinol. 19, 443–459. 10.1038/s41574-023-00845-0 37221402 PMC10204695

[B164] Zeng L. Yan J. (2025). Mechanisms of alveolar type II epithelial cells’ mitochondrial quality control during acute lung injury/acute respiratory distress syndrome: bridging the gap between oxidative stress, inflammation, and fibrosis. Front. Physiol. 16, 1684729. 10.3389/fphys.2025.1684729 41178984 PMC12571842

[B165] Zhang Y. Michelakis E. D. (2021). A phase-2 NIH-sponsored randomized clinical trial of rituximab in scleroderma-associated pulmonary arterial hypertension did not reach significance for its endpoints: end of story? Not so fast. Am. J. Respir. Crit. Care Med. 204, 123–125. 10.1164/rccm.202103-0612ED PMC8650784 33856964

[B166] Zhang X. Xu W. Xu R. Wang Z. Zhang X. Wang P. (2022). Plin5 bidirectionally regulates lipid metabolism in oxidative tissues. Oxid. Med. Cell Longev. 2022, 4594956. 10.1155/2022/4594956 35401929 PMC8989587

[B167] Zhang C. Zheng M. Bai R. Chen J. Yang H. Luo G. (2024). Molecular mechanisms of lipid droplets-mitochondria coupling in obesity and metabolic syndrome: insights and pharmacological implications. Front. Physiol. 15, 1491815. 10.3389/fphys.2024.1491815 39588271 PMC11586377

[B168] Zhang P. Zhang M. Liu J. Zhou Z. Zhang L. Luo P. (2025). Mitochondrial pathway signature (MitoPS) predicts immunotherapy response and reveals NDUFB10 as a key immune regulator in lung adenocarcinoma. J. Immunother. Cancer 13, e012069. 10.1136/jitc-2025-012069 40744665 PMC12315039

[B169] Zhu Y. Choi D. Somanath P. R. Zhang D. (2024a). Lipid-laden macrophages in pulmonary diseases. Cells 13, 889. 10.3390/cells13110889 PMC11171561 38891022

[B170] Zhu L. Wu Z. Liu Y. Ming Y. Xie P. Jiang M. (2024b). Acod1/itaconate activates Nrf2 in pulmonary microvascular endothelial cells to protect against the obesity-induced pulmonary microvascular endotheliopathy. Respir. Res. 25, 205. 10.1186/s12931-024-02827-w 38730297 PMC11088094

[B171] Zhuang Y. Zhang Z. Dai Z. Shi X. (2026). Landscape expansion microscopy reveals interactions between membrane and phase-separated organelles. J. Cell Biol. 225, e202502035. 10.1083/jcb.202502035 PMC12839970 41591359

[B172] Zimmermann C. M. Baldassi D. Chan K. Adams N. B. P. Neumann A. Porras-Gonzalez D. L. (2022). Spray drying siRNA-lipid nanoparticles for dry powder pulmonary delivery. J. Control Release 351, 137–150. 10.1016/j.jconrel.2022.09.021 36126785 PMC7613708

[B173] Zong Y. Li H. Liao P. Chen L. Pan Y. Zheng Y. (2024). Mitochondrial dysfunction: mechanisms and advances in therapy. Signal Transduct. Target Ther. 9, 124. 10.1038/s41392-024-01839-8 38744846 PMC11094169

